# Polyphenols from the Root Barks of *Ulmus davidiana* var. *japonica* and Their Wound Healing Potential

**DOI:** 10.3390/ijms27198630

**Published:** 2026-09-26

**Authors:** Jae Sik Yu, Gakyung Lee, Sooyeon Kim, Yoon-Joo Ko, Min Cheol Kang, Sun Yeou Kim, Min Hye Yang, Ki Hyun Kim

**Affiliations:** 1 Department of Integrative Biological Sciences and Industry, Sejong University, 209 Neungdong-ro, Gwangjin-gu, Seoul 05006, Republic of Korea; jsyu@sejong.ac.kr (J.S.Y.); lgg1025@sejong.ac.kr (G.L.); for_studying@naver.com (S.K.); 2 School of Pharmacy, Sungkyunkwan University, Suwon 16419, Republic of Korea; 3 Laboratory of Nuclear Magnetic Resonance, National Center for Inter-University Research Facilities (NCIRF), Seoul National University, Gwanak-gu, Seoul 08826, Republic of Korea; yjko@snu.ac.kr; 4 College of Pharmacy, Gachon University, Incheon 21936, Republic of Korea; mckang@metacen.com (M.C.K.); sunnykim@gachon.ac.kr (S.Y.K.); 5 Department of Pharmacy, College of Pharmacy and Research Institute for Drug Development, Pusan National University, Busan 46241, Republic of Korea

**Keywords:** *Ulmus davidiana* var. *japonica*, catechin glycoside derivative, neolignane derivatives, NMR, skin keratinocytes, skin wound-healing

## Abstract

The root bark of *Ulmus davidiana* var. *japonica* (Ulmaceae), commonly known as yugeunpi, has long been used in traditional Korean medicine to treat gastroenteric and inflammatory disorders, as well as to promote blood circulation and tissue regeneration. As part of our ongoing efforts to discover bioactive natural products from resources with pharmacological potential, a phytochemical investigation of the root bark of *U. davidiana* var. *japonica* was conducted, and the wound-healing potential of the isolated compounds was evaluated. This study led to the isolation of one new catechin glycoside derivative, (–)-catechinulmusol (**1**) and two new neolignan derivatives, neolignulmusides A and B (**2** and **3**), along with ten known compounds (**4**–**13**), including lignans (**4** and **5**), flavonoids (**6**–**10**), and phenolic derivatives (**11**–**13**). The structures of the new compounds (**1**–**3**) were elucidated by extensive spectroscopic analyses, including 1D and 2D NMR, high-resolution electrospray ionization mass spectrometry (HR-ESI-MS), and chemical derivatization. Results from in vitro assays revealed that compounds **1**, **4**, **6**, and **13** significantly promoted the migration of human skin keratinocytes. These findings suggest that these active compounds may serve as promising candidates for skin wound-healing applications, including epidermal regeneration.

## 1. Introduction

The skin serves as the body’s first line of defense against harmful external factors such as pathogens, chemicals, and physical stresses [1]. As important mediators of the immune system, keratinocytes and other skin cells play essential roles in initiating, maintaining, and amplifying inflammatory responses to facilitate the healing and restoration of damaged tissues [2]. Because the skin is constantly exposed to various insults, proper protection and treatment are vital for maintaining its integrity and function.

Wounds can occur as a result of numerous stimuli, including physical, chemical, electrical, or thermal injury. When the skin is damaged, it loses its protective barrier function, rendering the body susceptible to invasion by pathogenic microorganisms [3]. Wound healing is a highly intricate and well-orchestrated biological process through which the skin or tissue repairs itself after injury, driven by the body’s hemostatic and immune systems. This process proceeds through four overlapping stages: hemostasis, inflammation, proliferation, and tissue remodeling [4]. During these dynamic and interconnected phases, wound healing involves a cascade of cellular and biochemical events mediated by diverse cytokines, growth factors, and enzymes. Among these stages, inflammation and tissue remodeling are particularly crucial, as they prevent bacterial infection and promote tissue repair. Consequently, many studies have focused on improving wound management, especially during these phases. Various therapeutic agents, including antibiotics and nonsteroidal anti-inflammatory drugs (NSAIDs), are currently used in wound care. However, most of these therapies are associated with undesirable side effects. As a result, there has been growing interest in alternative approaches, such as traditional medicines and natural products. Several plant-derived extracts have long been used in wound dressings to accelerate the healing process, offering a potential source of safer and more effective treatments.

*Ulmus davidiana* var. *japonica* (Ulmaceae), commonly known as the Japanese elm tree, has a long history of use in oriental medicine. In Korea, the root bark, referred to as “yugeunpi,” has traditionally been used to treat inflammation, eczema, burns, skin allergies, and psoriasis [5,6,7,8]. In addition, it has been employed to alleviate gastroenteric and inflammatory disorders, promote blood circulation, and support the regeneration of damaged tissues [9]. Previous studies have reported various bioactivities of extracts and secondary metabolites from this plant, supporting its medicinal value. Steroids and catechin glycosides isolated from the root bark demonstrated inhibitory effects on eicosanoid generation and exhibited antiseptic properties by modulating inflammatory mediators such as TNF-α and IL-10 [10,11,12]. Furthermore, the methanolic extract of this plant was shown to reduce immunoglobulin and IgG2a levels in a mouse model of contact dermatitis [5]. When applied externally to wound sites in vivo, the root bark powder provided protection against secondary infections [13]. Similarly, the root bark powder of a related species, *U. parvifolia*, promoted skin wound healing by upregulating matrix metalloproteinase (MMP) and transforming growth factor-β (TGF-β) expression [14]. However, the specific bioactive compounds responsible for the wound-healing effects of *U. davidiana* var. *japonica* root bark have been rarely investigated. Therefore, it is important to identify and characterize these wound-healing constituents to scientifically validate the plant’s traditional use in supporting tissue regeneration.

As part of our ongoing efforts to discover bioactive natural products from resources with pharmacological potential [15,16,17,18,19,20], we conducted a phytochemical investigation to identify potential wound-healing compounds from the root bark of *U. davidiana* var. *japonica*. Phytochemical analysis of the ethanol (EtOH) extract, guided by liquid chromatography–mass spectrometry (LC–MS), led to the isolation of 13 compounds from the ethyl acetate (EtOAc)-soluble fraction, including one new catechin glycoside derivative (**1**) and two new neolignan derivatives (**2** and **3**). The chemical structures of the isolated compounds were elucidated using extensive spectroscopic analyses, including nuclear magnetic resonance (NMR), high-resolution electrospray ionization mass spectrometry (HR-ESI-MS), and chemical derivatization. Furthermore, the wound-healing potential of the isolated compounds was evaluated through a cell migration assay using HaCaT keratinocytes. Herein, we report the isolation, structural characterization, and wound-healing activity of these compounds from the root bark of *U. davidiana* var. *japonica*.

## 2. Results and Discussion

### 2.1. Isolation of Compounds

The EtOAc-soluble fraction, obtained from the EtOH extract of *U. davidiana* var. *japonica* root bark after sequential solvent partitioning with four organic solvents, was subjected to repeated column chromatography over Sephadex LH-20, followed by semi-preparative HPLC purification under LC–MS guidance, resulting in the isolation of thirteen phytochemicals (**1**–**13**). These were identified as one new catechin glycoside derivative (**1**) and two new neolignan derivatives (**2** and **3**), along with ten known compounds (**4**–**13**), which included flavonoid glycosides, lignans, and phenolic derivatives (Figure 1).

The known compounds were identified as icariside E4 (**4**) [21], (−)-9′-O-(α-L-rhamnopyranosyl)lyoniresinol (**5**) [22], isoquercetin (**6**) [23], isohemiphloin (**7**) [24], quercetin-4′-O-β-D-glucopyranoside (**8**) [25], hemiphloin (**9**) [26], 5,7-di-O-methyldihydroquercetin (**10**) [27], (2*R*,3*S*)-2-ethoxychroman-3,5,7-triol-7-O-β-D-apiofuranoside (**11**) [28], 5-hydroxl,4-methoxy-3-O-β-D-glucose benzoic acid (**12**) [29], and 2-[4-(3-hydroxypropyl)-2-methoxyphenoxyl]-1,3-propanediol (**13**) [30] through comparison of their NMR spectroscopic data with previously reported values, as well as LC–MS data.

### 2.2. Structural Elucidation of New Compounds

Compound **1** was isolated as a yellowish gum and its molecular formula was identified as C_31_H_34_O_13_ (15 degrees of unsaturation) by the information from the positive-ion HR-ESI-MS data at *m*/*z* 615.2072 [M + H]^+^ (calc’d for C_31_H_35_O_13_ 615.2078, Δ −0.98 ppm) and NMR data (Table 1). The ^1^H NMR spectrum of **1** (Table 1) combined with the HSQC experiment showed signals for set of aromatic proton signals at *δ*_H_ 6.22 (1H, d, *J* = 2.0 Hz, H-6), 6.26 (1H, d, *J* = 2.0 Hz, H-8), 6.75 (1H, dd, *J* = 8.0, 2.0 Hz, H-6′), 6.77 (1H, d, *J* = 8.0 Hz, H-5′), 6.86 (1H, d, *J* = 2.0 Hz, H-2′), 7.54 (2H, d, *J* = 8.5 Hz, H-9″/H-13″), and 8.00 (2H, d, *J* = 8.5 Hz, H-10″/H-12″). Among these signals, one aromatic ring exhibited *meta*-coupled proton resonances at *δ*_H_ 6.22 and 6.26, while proton signals at *δ*_H_ 6.75, 6.77, and 6.86 corresponded to another aromatic ring, characteristic of a 1,3,4-trisubstituted benzene moiety, based on their coupling constants. In addition, the resonances at *δ*_H_ 7.54 and 8.00 were attributed to a 1,4-substituted benzene ring. In addition, the presence of two oxygenated methine groups was confirmed from the signals at *δ*_H_ 4.72 (1H, d, *J* = 6.5 Hz)/*δ*_C_ 81.1 and *δ*_H_ 4.02 (1H, m)/*δ*_C_ 67.3, two methyl signals were present at *δ*_H_ 1.53 (3H, s, H-15″) and 1.52 (3H, s, H-16″), and finally one methylene group can be deduced from *δ*_H_ 2.83 (1H, ddd, *J* = 16.5, 5.0 Hz) and 2.59 (1H, ddd, *J* = 16.5, 7.5 Hz)/*δ*_C_ 26.2. Furthermore, the presence of a glucopyranose moiety was indicated by an anomeric proton signal at *δ*_H_ 4.92 (1H, d, *J* = 7.5 Hz, H-1″)/*δ*_C_ 100.5, along with characteristic proton resonances *δ*_H_ 4.72 (1H, overlap, H-6a″), 4.36 (1H, dd, *J* = 12.0, 6.5 Hz, H-6b″), 3.79 (1H, m, H-5″), 3.51 (1H, m, H-3″), 3.47 (1H, m, H-4″), and 3.47 (1H, m, H-2″), together with the corresponding carbon resonances at *δ*_C_ 76.3, 73.9, 73.1, 70.1, and 63.9 [31]. The coupling constant (*J* = 7.5 Hz) of the anomeric proton at *δ*_H_ 4.92 matched well with the sugar moiety as *β*-glucopyranose [32].

The planar structure of **1** was fully elucidated with the assistance of COSY and HMBC analysis (Figure 2). The COSY spectrum indicated the correlations starting from oxygenated methine *δ*_H_ 4.72 (H-2) via *δ*_H_ 4.02 (H-3) and ending at *δ*_H_ 2.83 and 2.59 (H_2_-4). The HMBC correlations of *δ*_H_ 4.92 (H-1″) to *δ*_C_ 157.2 (C-7) showed that the β-glucopyranose moiety was linked to C-7. Comprehensive analysis of the NMR chemical shifts and 2D NMR correlations revealed a close resemblance to those of previously reported (–)-catechin 7-*O*-β-D-glucopyranoside [33]. Furthermore, the HMBC spectrum showed that the aromatic proton signals at *δ*_H_ 8.00 (H-10″/H-12″) of the 1,4-substituted benzene ring exhibited correlations with the carbon resonance at *δ*_C_ 166.7 (C-14″), indicating the presence of a carboxyl group at C-11″. Another pair of aromatic proton signals at *δ*_H_ 7.54 (H-9″/H-13″) displayed correlations with a quaternary carbon at *δ*_C_ 71.5 (C-7″). This quaternary carbon at *δ*_C_ 71.5 (C-7″) also showed correlations with methyl proton signals at *δ*_H_ 1.53 (H-15″) and 1.52 (H-16″). These overall correlations defined a 4-(1-hydroxy-1-methylethyl)benzoic acid moiety [34]. Additionally, the proton signals at *δ*_H_ 4.72 and 4.36 (H_2_-6″) of the β-glucopyranose moiety exhibited HMBC correlations to the quaternary carbon at *δ*_C_ 71.5 (C-7″) of the 4-(1-hydroxy-1-methylethyl)benzoic acid unit. This correlation explains the unusual downfield shift in the H-6″ protons, which is attributed to the attachment of the 4-(1-hydroxy-1-methylethyl)benzoic acid moiety to C-6″ through the oxygen atom of C-7″ (Figure 2).

To determine the absolute configuration of **1**, acid hydrolysis was performed, affording the catechin aglycone, *β*-glucopyranose, and 4-(1-hydroxy-1-methylethyl)benzoic acid. The 2,3-*trans* configuration of **1** was confirmed by the characteristic coupling constant (*J* = 6.5 Hz) observed between H-2 and H-3 in the ^1^H NMR data [35]. The catechin aglycone obtained from acid hydrolysis was further characterized by its optical rotation and electronic circular dichroism (ECD) spectrum. The optical rotation (${\left[a\right]}_{D}^{25}-22.0$) and ECD spectrum of the catechin aglycone, including a negative Cotton effect at 230 nm (*Δ*ε = −0.60) and a positive Cotton effect at 270 nm (*Δ*ε = +0.28) (Figure S10), were consistent with the reported spectroscopic characteristics of (−)-catechin, supporting the assignment of its absolute configuration [36]. To establish the absolute configuration of the sugar moiety, *β*-glucopyranose obtained from acid hydrolysis of compound **1** and an authentic *β*-D-glucopyranose standard was derivatized to the corresponding thiocarbamoyl-thiazolidine derivatives following the reported method [37]. The retention times of the resulting derivatives were compared by LC–MS analysis, which revealed that the *β*-glucopyranose from compound **1** was of the D-configuration. Therefore, the complete structure of **1** was elucidated as shown in Figure 1 and designated as (–)-catechinulmusol.

Compound **2** was isolated as a colorless gum with the molecular formula of C_27_H_36_O_11_ (10 degrees of unsaturation), which was determined from the positive-ion HR-ESI-MS data at *m*/*z* 537.2328 [M + H]^+^ (calc’d for C_27_H_37_O_11_ 537.2336, Δ −1.49 ppm) and NMR data in CD_3_OD (Table 1). The ^1^H NMR spectrum of **2** (Table 1) combined with the HSQC experiment showed the presence of aromatic signals at *δ*_H_ 6.74 (1H, brs, H-6′), 6.72 (1H, brs, H-2′), and 6.72 (2H, s, H-2/H-6), which represented two 1,3,4,5-tetrasubsituted aromatic rings as well as one oxygenated methine at *δ*_H_ 5.56 (1H, d, *J* = 6.0 Hz, H-7), two oxygenated methylenes at *δ*_H_ 3.86 (1H, overlap, H-9a), 3.76 (1H, dd, *J* = 11.0, 7.5 Hz, H-9b), and 3.57 (2H, t, *J* = 6.5 Hz, H-9′), one methine *δ*_H_ 3.46 (1H, m, H-8), two methylenes at *δ*_H_ 2.73 (2H, m, H-7′) and 1.82 (2H, m, H-8′), and three methoxy groups at *δ*_H_ 3.87 (3H, s, 3′-OCH_3_) and *δ*_H_ 3.80 (6H, s, 5-OCH_3_/3-OCH_3_). In addition, the presence of an α-rhamnopyranosyl unit was indicated by the anomeric proton at *δ*_H_ 5.21 (1H, d, *J* = 1.0 Hz, H-1″)/*δ*_C_ 103.3, the methyl group at *δ*_H_ 1.20 (1H, d, *J* = 6.0 Hz, H-6″)/*δ*_C_ 17.6, and four oxygenated methine signals at *δ*_H_ 4.29 (H-5″)/*δ*_C_ 70.8, *δ*_H_ 4.11 (H-2″)/*δ*_C_ 71.8, *δ*_H_ 3.87 (H-3″)/*δ*_C_ 72.0, and *δ*_H_ 3.42 (H-4″)/*δ*_C_ 73.5 [38]. The overall NMR data closely resembled those of the previously reported neolignan derivative, chaenomiside A [38]. However, a notable difference was observed in the anomeric proton of the rhamnopyranosyl unit, which appeared deshielded at *δ*_H_ 5.21/*δ*_C_ 103.3, compared to chaenomiside A. The planar structure of the compound **2** was further confirmed by 2D NMR analyses, including HMBC, HSQC, and COSY experiments (Figure 3). Guided by the HMBC analysis, a clear correlation between H-1″ and C-4 was observed, indicating that the rhamnopyranosyl unit is linked at C-4. This linkage explains the downfield shift in the anomeric proton (*δ*_H_ 5.21) compared to that of chaenomiside A (*δ*_H_ 4.77). To determine the absolute configuration of **2**, acid hydrolysis was carried out, affording the aglycone and a rhamnopyranose unit. The rhamnopyranose was identified based on its specific optical rotation, (${\left[a\right]}_{D}^{25}+10.5$), and subsequent chemical derivatization, which confirmed the presence of L-rhamnose. In addition, the coupling constant of the anomeric proton (*J* = 1.0 Hz) indicated an α-L-rhamnopyranosyl configuration [38]. The relative configuration of C-7 and C-8 was established as *trans* from the coupling constant (*J* = 6.0 Hz), consistent with literature data [38,39]. The absolute configuration of C-7 and C-8 was determined as 7*R* and 8*S*, respectively, based on the positive Cotton effect at 226 nm and negative Cotton effects at 241 and 290 nm observed in the experimental ECD spectrum of the lignan aglycone [40]. Therefore, the structure of **2** was elucidated as shown in Figure 1 and designated as neolignulmuside A.

Compound **3** was isolated as a colorless gum with the molecular formula of C_23_H_30_O_9_ (9 degrees of unsaturation), as determined from the positive-ion HR-ESI-MS data at *m*/*z* 451.1955 [M + H]^+^ (calc’d for C_23_H_31_O_9_ 451.1968, Δ −2.88 ppm) and NMR data (Table 1). The ^1^H NMR spectrum of **3** (Table 1) combined with the HSQC experiment showed the presence of aromatic signals at *δ*_H_ 6.77 (2H, s, H-2/H-6), 6.59 (1H, brs, H-6′), and 6.58 (1H, s, H-2′), which represented the two 1,3,4,5-tetrasubstituted aromatic rings as well as two oxygenated methine at *δ*_H_ 5.57 (1H, d, *J* = 5.5 Hz, H-7) and 3.99 (1H, quint, *J* = 5.0 Hz, H-2″), four oxygenated methylenes at *δ*_H_ 3.87 (1H, dd, *J* = 11.0, 5.0, H-9a), 3.75 (1H, m, H-9b), 3.74 (4H, dd, *J* = 5.0, 1.5, H-1″/H-3″) and 3.55 (2H, t, *J* = 6.5 Hz, H-9′), one methine *δ*_H_ 3.44 (1H, m, H-8), two methylenes at *δ*_H_ 2.56 (2H, m, H-7′) and 1.79 (2H, m, H-8′), and two methoxy groups at *δ*_H_ 3.83 (6H, s, 5-OCH_3_/3-OCH_3_). Characteristic resonances at *δ*_H_ 3.99/*δ*_C_ 84.6 and 3.74/*δ*_C_ 61.9 indicated the presence of a glycerol unit in the structure [41]. Based on 2D NMR analyses (HMBC, HSQC, and COSY), the overall spectral features closely resembled those of compound **2** (Figure 4). However, unlike compound **2**, compound **3** lacks a methoxy substituent at C-3′, and instead of a rhamnopyranosyl group linked at C-4, a glycerol unit is attached at this position (Figure 4). To determine the absolute configuration of **3**, the same approach used for compound **2** was applied. The relative configuration of C-7 and C-8 was established as *trans* based on the coupling constant (*J* = 5.5 Hz), consistent with reported data [38,39]. The absolute configuration of the dihydrobenzofuran moiety of **3** was assigned as 7*S*,8*R* based on its experimental ECD spectrum, which exhibited a negative Cotton effect at 235 nm and positive Cotton effects at 254 and 290 nm, consistent with the reported ECD characteristics of structurally related dihydrobenzofuran-type neolignans [40]. Although the glycerol substituent contains an additional stereogenic center at C-2″, it does not possess an independent UV-active chromophore, and the observed Cotton-effect pattern is considered to arise predominantly from the aromatic dihydrobenzofuran chromophore. Nevertheless, a possible influence of the glycerol substituent on the ECD spectrum cannot be entirely excluded. On the basis of the experimental ECD data, the established *trans* relative configuration of C-7/C-8, and comparison with literature data, the 7*S*,8*R* configuration was assigned to the dihydrobenzofuran moiety of **3**, which was designated as neolignulmuside B.

### 2.3. Cell Migration Assay

To investigate whether the isolated compounds contribute to the rejuvenation of wound repair, the rate of cell migration was assessed by measuring the percentage of wound closure across the induced gap in the scratch assay. Prior to the migration assay, the cytotoxicity of compounds **1**–**13** was evaluated in HaCaT cells at 10 μM. None of the tested compounds showed significant cytotoxicity under these experimental conditions (Figure S29). As shown in Figure 5, compounds **1**, **4**, **6**, and **13** significantly enhanced wound closure, indicating accelerated migration and proliferation of HaCaT keratinocytes. Notably, the wound-healing activity of isoquercetin (**6**) observed in the present study is consistent with previous findings. Our recent study demonstrated that isoquercetin at 10 μM promoted wound closure in a HaCaT cell scratch assay, providing direct evidence for its ability to enhance keratinocyte migration [42]. Furthermore, Bhatia et al. demonstrated the wound-healing activity of isoquercetin in vivo using a rat scald-burn model, in which topical treatment with isoquercetin-based cream significantly enhanced wound contraction and shortened the epithelialization period [43]. Histological examination further revealed improved epidermal regeneration and re-epithelialization, accompanied by decreased TBARS and restored GSH levels in the wound tissue [43]. Taken together, these previous in vitro and in vivo findings support the wound-repair activity of isoquercetin observed in our study and suggest that its promotion of keratinocyte migration may contribute to re-epithelialization, potentially in conjunction with attenuation of oxidative and inflammatory tissue damage.

## 3. Materials and Methods

### 3.1. General Experimental Procedures

Optical rotations were measured in MeOH using a Jasco P-2000 polarimeter (Jasco, Easton, MD, USA). Infrared (IR) spectra were recorded on an IFS-66/s FT-IR spectrometer (Bruker, Karlsruhe, Germany). Electronic circular dichroism (ECD) spectra were recorded in MeOH using a JASCO J-1500 spectropolarimeter (JASCO, Tokyo, Japan) with a quartz cuvette (1 mm optical path length). Ultraviolet (UV) spectra were acquired on an Agilent 8453 UV-visible spectrophotometer (Agilent Technologies, Santa Clara, CA, USA). High-resolution electrospray ionization mass spectra (HRESIMS) were recorded on Waters Xevo G2 QTOF mass spectrometer (Waters Co., Milford, MA, USA) and Synapt G2 HDMS quadrupole time-of-flight (TOF) mass spectrometer (Waters Co., Milford, MA, USA). All NMR experiments were performed on an 800 MHz NMR spectrometer (Bruker, Karlsruhe, Germany) equipped with a TCI CryoProbe. Samples were dissolved in CD_3_OD, and spectra were acquired at approximately 298 K. The ^1^H and ^13^C resonance frequencies were 800.190 and 201.208 MHz, respectively. Gradient-selected, multiplicity-edited ^1^H–^13^C HSQC spectra were acquired using the *hsqcedetgpsisp2.3* pulse sequence, with the INEPT transfer optimized for an average one-bond coupling constant (^1^*J*_CH_) of 145 Hz. The acquisition matrix consisted of 2048 × 400 data points in the ^1^H and ^13^C dimensions, respectively, with a relaxation delay of 1.0 s. ^1^H–^13^C HMBC spectra were acquired using the *hmbcgplpndqf* pulse sequence, with the long-range transfer optimized for a coupling constant (^n^*J*_CH_) of 8 Hz and the one-bond coupling constant (^1^*J*_CH_) set to 145 Hz. Depending on the sample, the acquisition matrix consisted of 2048 × 400 or 2048 × 256 data points in the ^1^H and ^13^C dimensions, respectively, with a relaxation delay of 1.5 s. Phase-sensitive NOESY spectra were acquired using the *noesyphpp.2* pulse sequence with a mixing time of 300 ms and a relaxation delay of 1.0 s. Chemical shifts (δ) were referenced to the residual solvent signals of CD_3_OD (δ_H_ 3.31 and δ_C_ 49.0 ppm). Preparative high-performance liquid chromatography (HPLC) was conducted using a Waters 1525 binary HPLC pump with Waters 996 photodiode array detector (Waters Corporation, Milford, CT, USA). Semi-preparative HPLC was conducted with a Shimadzu Prominence HPLC System with SPD-20A/20AV series Prominence HPLC UV-Vis detectors (Shimadzu, Tokyo, Japan). The solvent systems and elution conditions used for column chromatography and preparative HPLC are specified in the corresponding extraction and isolation procedures. LC/MS analysis was performed on an Agilent 1200 series HPLC system with a diode array detector and 6130 Series ESI mass spectrometer using an analytical Kinetex C_18_ 100 Å column (100 mm × 2.1 mm i.d., 5 μm) (Phenomenex, Torrance, CA, USA). Column chromatography used silica gel 60, 230–400 mesh, RP-C_18_ silica gel, 230–400 mesh (Merck, Darmstadt, Germany). The packing material for molecular sieve column chromatography was Sephadex LH-20 (Pharmacia, Uppsala, Sweden). Diaion HP-20 (Mitsubishi Chemical, Tokyo, Japan) was also used for open-column chromatography. Thin-layer chromatography (TLC) was conducted using precoated silica gel F_254_ plates and reverse-phase (RP)-C_18_ F_254s_ plates (Merck), using hexanes, dichloromethane, ethyl acetate, methanol and water as developing solvents. Spots on TLC were detected using UV and heating after dipping in anisaldehyde-sulfuric acid.

### 3.2. Plant Material

The root barks of *U*. *davidiana* var. *japonica* were collected in Wonhwasan-ro, Jecheon-si, Chungcheongbuk-do, Korea, in 2016 and purchased by Donggwang General Corporation. The plant was authenticated by one of the authors (K.H. Kim). A voucher specimen (SKKU-NR 0401) of the plant has been deposited at the herbarium of the School of Pharmacy, Sungkyunkwan University, Suwon, Korea.

### 3.3. Extraction and Isolation

The dried root barks of *U*. *davidiana* var. *japonica* (10 kg) were extracted with 50% aqueous EtOH (each 60 L × 2 days) at 70 °C and filtered. The filtrate was combined and concentrated under reduced pressure using a rotavapor to obtain the EtOH extract (900 g). The EtOH extract (300 g) was divided in half and suspended in distilled water (800 mL) each, and then was successively solvent-partitioned with hexane, dichloromethane (CH_2_Cl_2_), ethyl acetate (EtOAc) and *n*-butanol (BuOH). Four layers with increasing polarity, hexane-soluble (4 g), CH_2_Cl_2_-soluble (39 g), EtOAc-soluble (25 g) and BuOH-soluble fractions (81 g) were obtained. By comparison with our house-built UV library, LC/MS analysis of the four fractions from the solvent partitioning indicated the presence of major phenolic compounds in the EtOAc-soluble fraction. The EtOAc-soluble fraction (25 g) was subjected to a Diaion HP-20 column in a gradient solvent system of MeOH (100% H_2_O, 20% MeOH, 40% MeOH, 60% MeOH, 80% MeOH, and 100% MeOH) to yield six fractions (E0, E2, E4, E6, E8 and E10). Fraction E4 (13.1 g) was subjected to silica gel column chromatography eluted with a gradient solvent system of CH_2_Cl_2_/MeOH (20:1 → 1:1) and 100% MeOH to provide five fractions (E41-E44). Among those fractions, fraction E43 (2.2 g) was further subjected to reversed phase C_18_ column chromatography to offer seven fractions (E431-E437). Subfraction E437 (98.4 mg) was fractionated by Sephadex LH-20 column chromatography using a solvent system of CH_2_Cl_2_/MeOH (5:1), which provided six fractions (E4371–E4376). Fraction E4373 (6.6 mg) was purified using semi-preparative HPLC with a 21–24% MeOH/H_2_O gradient system to give compound **7** (1.3 mg, *t*_R_ = 27.5 min). Fraction E434 (1.6 g) was fractionated using preparative reversed-phase HPLC with a gradient solvent system of MeOH/H_2_O (1:9 → 8:2) to provide four fractions (E4341–E4344). Subfraction E4342 (20.9 mg) was purified using semi-preparative HPLC with a 18% MeOH/H_2_O system to give compound **10** (3.3 mg, *t*_R_ = 21.0 min). Fraction E436 (24.2 mg) was isolated using semi-preparative HPLC with a 21–24% MeOH/H_2_O gradient system to afford compound **11** (2.2 mg, *t*_R_ = 53.0 min). Fraction E45 (3.5 g) was subjected to reversed phase C_18_ column chromatography to yield nine fractions (E451-E459). Subfraction E451 (31.0 mg) was separated using semi-preparative HPLC with a 30% MeCN/H_2_O system to purify compound **12** (0.4 mg, *t*_R_ = 47.0 min). Fraction E6 (2.6 g) was subjected to silica gel column chromatography eluted with a gradient solvent system of CH_2_Cl_2_/MeOH (50:1 → 1:1) and 100% MeOH to afford seven fractions (E61-E67). Fraction E61 (23.8 mg) was purified using semi-preparative HPLC with a 15% MeCN/H_2_O system to yield compound **13** (0.4 mg, *t*_R_ = 30.0 min). Fraction E63 (0.2 g) was subjected to preparative reversed-phase HPLC with a 45–75% MeOH/H_2_O gradient system to provide six subfractions (E631-E636). Subfraction E636 (4.6 mg) was further isolated with semi-preparative HPLC with a 10–30% MeCN/H_2_O gradient system to give compound **1** (1.4 mg, *t*_R_ = 60.0 min). Fraction E8 (1.1 g) was fractionated by reversed-phase HPLC with a 45–65% MeOH/H_2_O gradient system to afford seven fractions (E81–E87). Fraction E82 (192.1 mg) was further subjected to Sephadex LH-20 column chromatography with a 100% MeOH system to make four fractions (E821–E824). Compounds **3** (0.4 mg, *t*_R_ = 42.0 min) and **5** (5.4 mg, *t*_R_ = 43.0 min) were all purified from subfraction E821 (53.1 mg) using semi-preparative HPLC with a 34% MeOH/H_2_O system. Subfraction E824 (18.9 mg) was further isolated using semi-preparative HPLC with a 33% MeOH/H_2_O system to furnish compound **9** (1.8 mg, *t*_R_ = 45.0 min). Fraction E83 (228.5 mg) was separated using Sephadex LH-20 column chromatography with a 100% MeOH system to yield five fractions (E831-E835). Compounds **4** (1.3 mg, *t*_R_ = 42.0 min) and **2** (2.2 mg, *t*_R_ = 44.0 min) were isolated from the subfraction E831 (26.4 mg) by semi-preparative HPLC with a 40% MeOH/H_2_O system. Fraction E84 (103.8 mg) was fractionated using reversed-phase HPLC with a 24–45% MeOH/H_2_O gradient system to produce six fractions (E841–E846). Subfraction E841 (19.1 mg) was further purified with semi-preparative HPLC with a 15–25% MeCN/H_2_O gradient system, which resulted in the isolation of compound **8** (0.8 mg, *t*_R_ = 34.0 min). Finally, fraction E85 (65.4 mg) was further fractionated with Sephadex LH-20 column chromatography with a 100% MeOH system to yield five subfractions (8E51–8E55). Among them, subfraction 8E55 (12.3 mg) was isolated using semi-preparative HPLC with a 46% MeOH/H_2_O system to produce compound **6** (2.6 mg, *t*_R_ = 27.5 min).

#### 3.3.1. (–)-Catechinulmusol (1)

Yellowish gum; ${\left[\alpha \right]}_{D}^{25}$ − 41.0 (*c* 0.03, MeOH); UV (MeOH) λ_max_ (log ε) 268 (3.5), 210 (3.8) nm; ECD (MeOH) λ_max_ (*Δ*ε) 249 (−0.71), 270 (0.21), 290 (−0.42) nm; ^1^H (800 MHz, CD_3_OD) and ^13^C (200 MHz, CD_3_OD) NMR data, see Table 1; HR-ESIMS (positive-ion mode) *m*/*z*: 615.2072 [M + H]^+^ (calcd. for C_31_H_35_O_13_, 615.2078).

#### 3.3.2. Neolignulmuside A (2)

Colorless gum; ${\left[\alpha \right]}_{D}^{25}$ + 15.9 (*c* 0.11, MeOH); UV (MeOH) λ_max_ (log ε) 280 (2.6), 234 (3.8), 206 (1.6) nm; ECD (MeOH) λ_max_ (*Δ*ε) 225 (+0.05), 231 (−0.14), 290 (−0.07) nm; ^1^H (800 MHz, CD_3_OD) and ^13^C (200 MHz, CD_3_OD) NMR data, see Table 1; HR-ESIMS (positive-ion mode) *m*/*z*: 537.2328 [M + H]^+^ (calcd. for C_27_H_37_O_11_, 537.2336).

#### 3.3.3. Neolignulmuside B (3)

Colorless gum; ${\left[\alpha \right]}_{D}^{25}$ + 74.0 (*c* 0.02, MeOH); UV (MeOH) λ_max_ (log ε) 230 (2.2), 210 (3.8) nm; ECD (MeOH) λ_max_ (*Δ*ε) 235 (−0.16), 254 (+0.04), 270 (−0.08), 291 (+0.14) nm; ^1^H (800 MHz, CD_3_OD) and ^13^C (200 MHz, CD_3_OD) NMR data, see Table 1; HR-ESIMS (positive-ion mode) *m*/*z*: 451.1955 [M + H]^+^ (calcd. for C_23_H_31_O_9_, 451.1968).

### 3.4. Acid Hydrolysis of ***1*** and ***2***

The absolute configuration of the sugar moiety was determined following previously reported methods [37,44,45]. Compounds **1** (0.5 mg) and **2** (1.0 mg) were hydrolyzed in the presence of 1 N HCl at 80 °C for 2 h and EtOAc was used for extraction. The aqueous layer was neutralized with repeated evaporation under a vacuum evaporator and dissolved in anhydrous pyridine (0.5 mL) with addition of L-cysteine methyl ester hydrochloride (2 mg). After the reaction mixture was heated at 60 °C for 1.5 h, *o*-tolyl isothiocyanate (50 μL) was added and the mixture was kept at 60 °C for 1 h. The reaction product was evaporated under a vacuum evaporator and dissolved in MeOH. After then, the resulting reaction product was dissolved and directly analyzed by LC–MS, and its retention time was compared with those of authentic standards (Figures S9 and S19).

### 3.5. Cell Culture

Immortalized human keratinocytes (HaCaT) were obtained from the Korean Cell Line Bank (Seoul National University, Seoul, Korea). Cells were cultured in high-glucose DMEM (Thermo Scientific, Waltham, MA, USA) supplemented with 10% fetal bovine serum (FBS) and 1% penicillin–streptomycin. Cultures were maintained in a humidified incubator at 37 °C with 5% CO_2_.

### 3.6. Cell Migration Assay

HaCaT cells were seeded at a density of 4 × 10^4^ cells/well in 96-well plates one day before the assay. A uniform wound was generated using a WoundMaker™ tool (Essen BioScience, Ltd., Hertfordshire, UK). The cells were then treated with each compound at 10 μM in 100 μL of serum-free medium and incubated at 37 °C for 24 h. The compounds were dissolved in DMSO, and the final concentration of DMSO in the culture medium was 0.1% (*v*/*v*). Cells treated with 0.1% DMSO without test compounds served as the vehicle control. Wound closure was monitored using the IncuCyte ZOOM™ live-cell imaging system (Essen BioScience, Ltd., Hertfordshire, UK), with images acquired every 2 h for 24 h. The quantitative data shown in Figure 5 were based on wound closure measured at the 24 h endpoint.

### 3.7. Statistical Analysis

Differences between groups were analyzed using one-way ANOVA followed by Dunnett’s or Tukey’s post hoc test. A value of *p* < 0.05 was considered statistically significant. Data are expressed as the mean ± standard error of the mean (SEM) from three independent experiments. Statistical analyses were performed using GraphPad Prism 5 (GraphPad Software, Inc., La Jolla, CA, USA).

## 4. Conclusions

LC/MS-based phytochemical analysis of the ethanolic extract of the root bark of *U. davidiana* var. *japonica* led to the isolation of one new catechin glycoside derivative, (–)-catechinulmusol (**1**), and two new neolignan derivatives, neolignulmusides A and B (**2** and **3**), along with ten known compounds (**4**–**13**) including lignans, flavonoids, and phenolic derivatives. The wound-healing effects of the isolated compounds were evaluated using a scratch assay in HaCaT keratinocytes, revealing that compounds **1**, **4**, **6**, and **13** significantly enhanced wound closure rates. These findings suggest that bioactive phytochemicals from the root bark of *U. davidiana* var. *japonica* may serve as promising candidates for the treatment of cutaneous wound damage and skin regeneration.

## Figures and Tables

**Figure 1:**
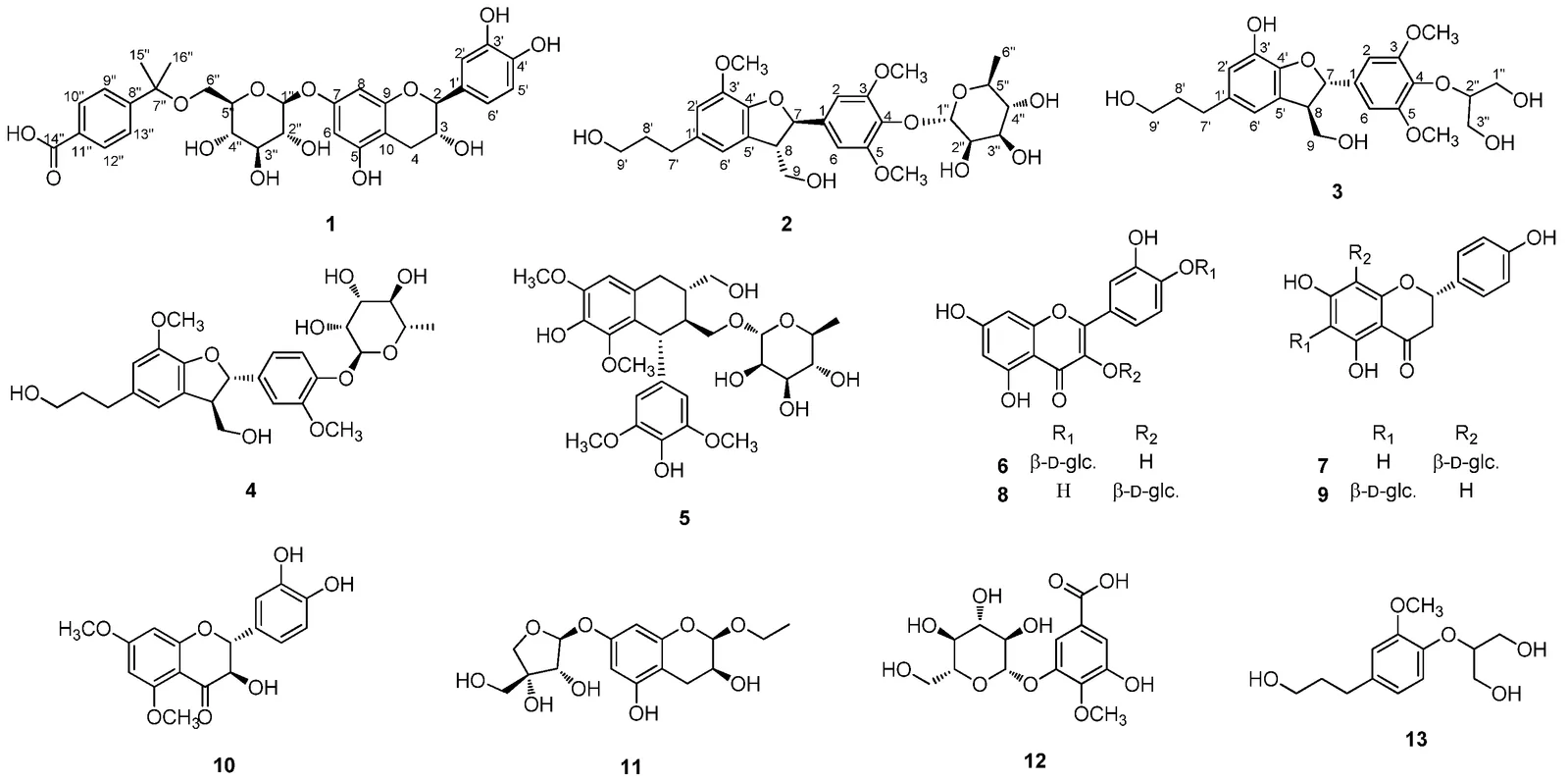
Chemical structures of compounds **1**–**13**.

**Figure 2:**
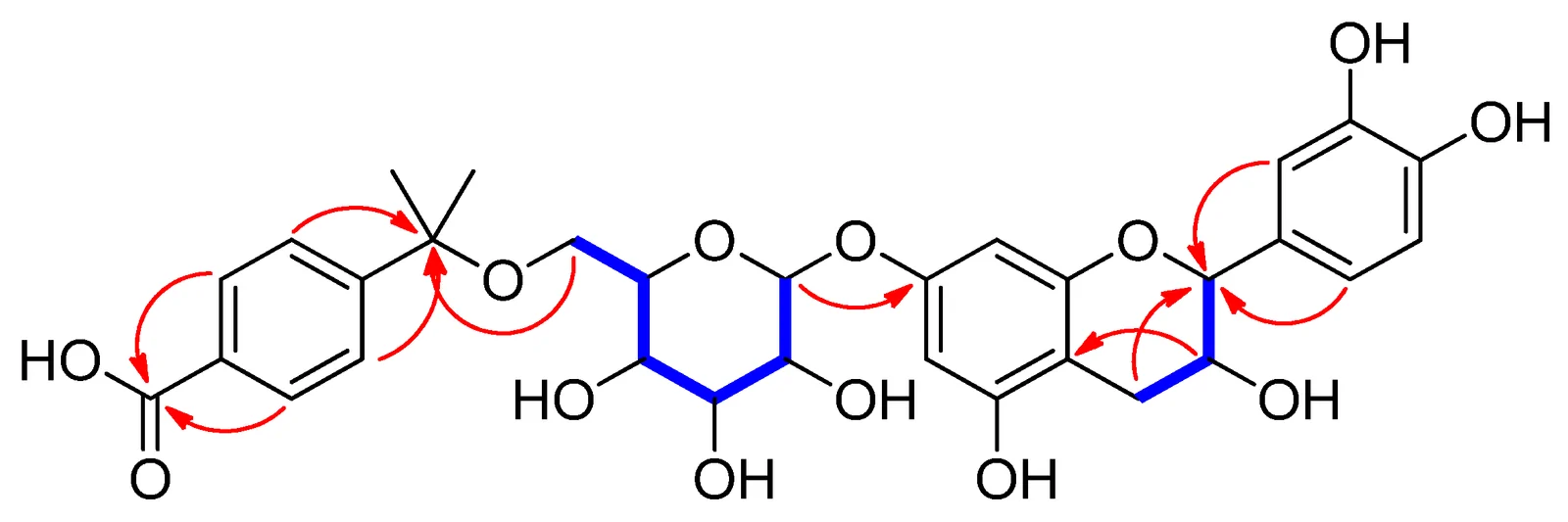
Key HMBC (red arrow) and COSY correlations (blue) of compound **1**.

**Figure 3:**
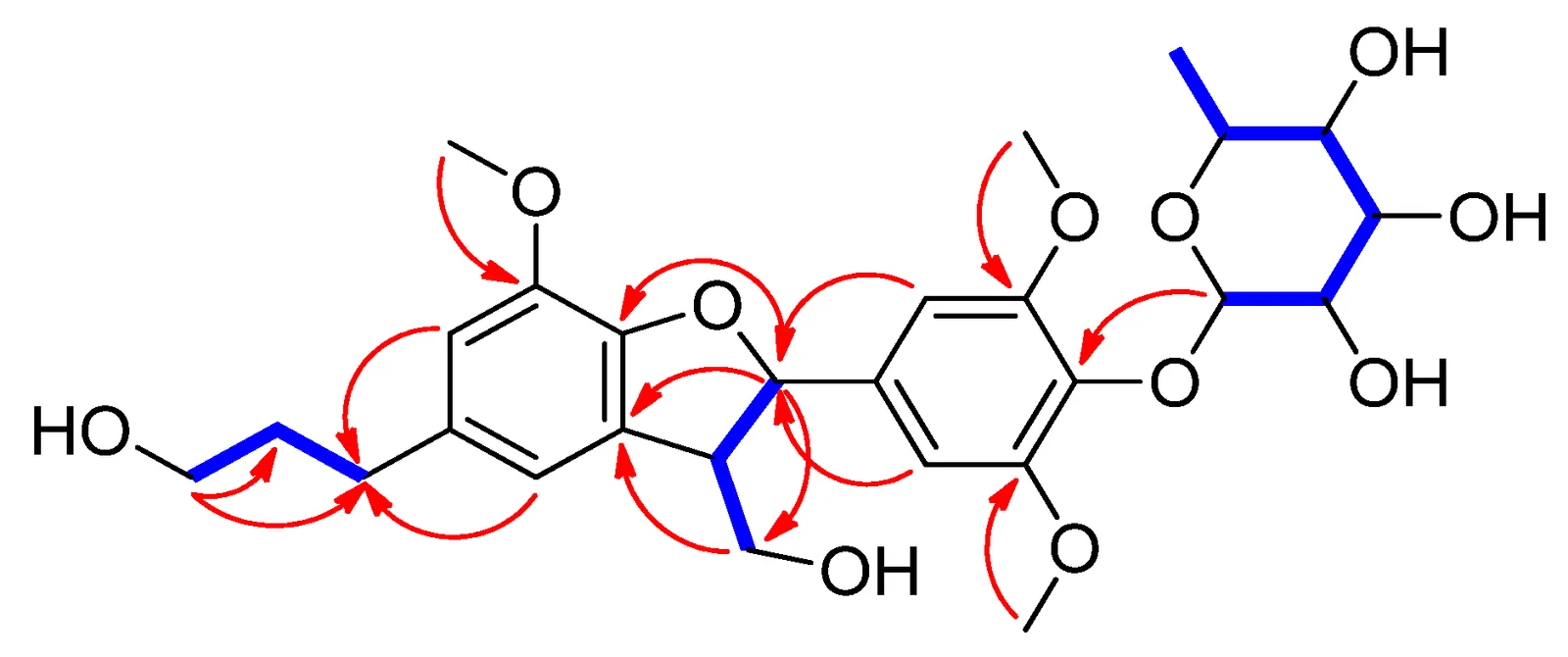
Key HMBC (red arrow) and COSY correlations (blue) of compound **2**.

**Figure 4:**
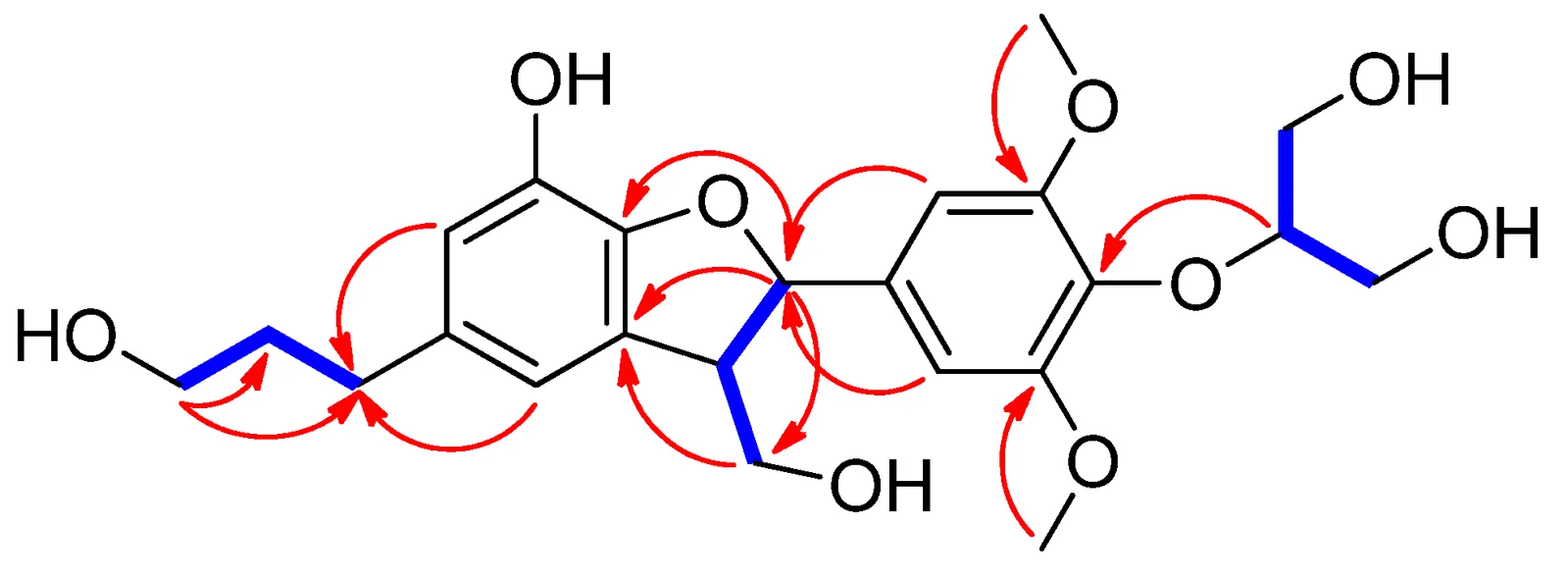
Key HMBC (red arrow) and COSY correlations (blue) of compound **3**.

**Figure 5:**
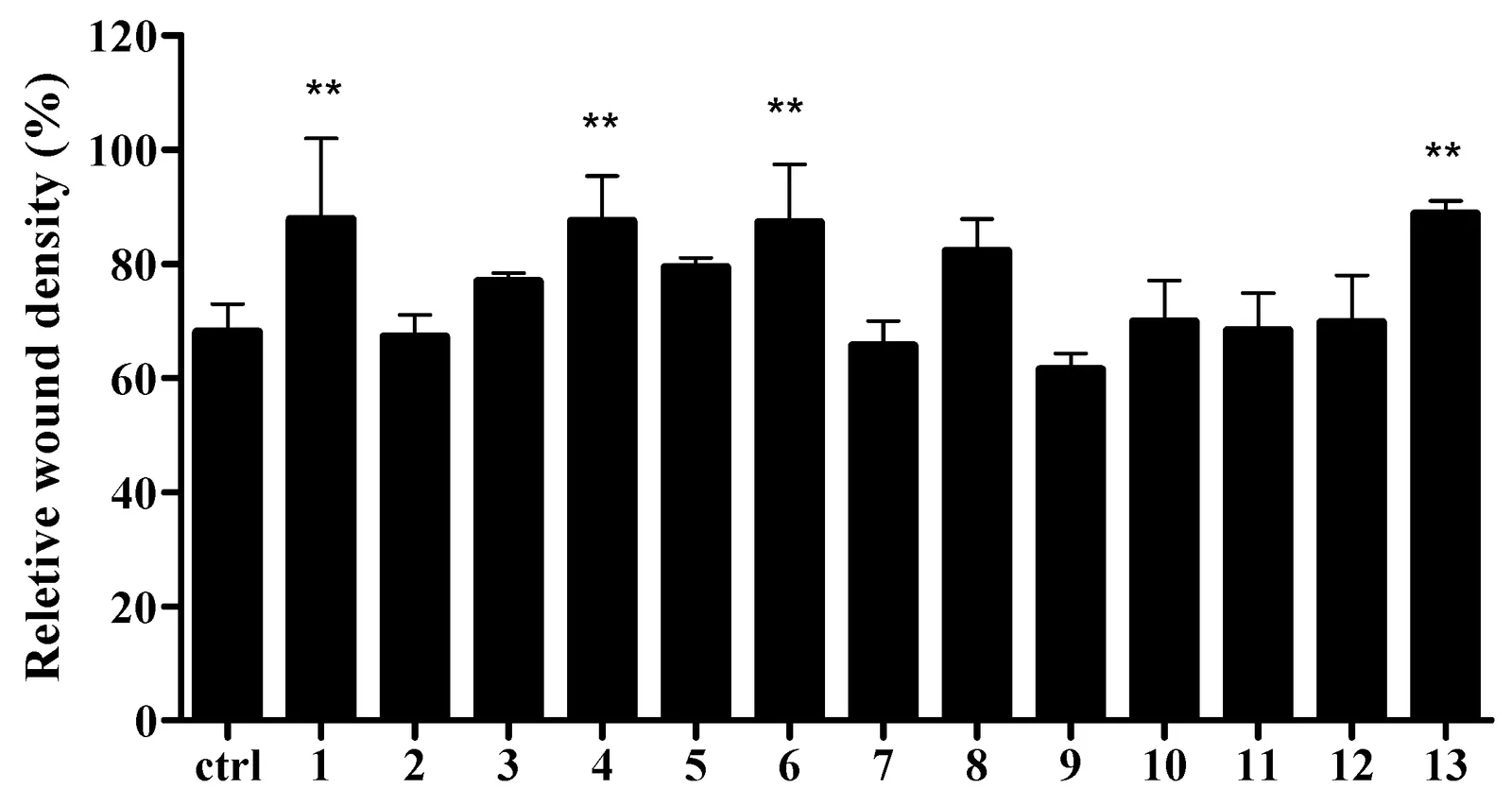
Effects of isolated compounds on migration in HaCaT cells. Effects of isolated compounds on the migration of HaCaT cells. Cells were cultured in 96-well plates and treated with each isolated compound (10 µM). Wound closure was monitored every 2 h using the IncuCyte ZOOM™ live-cell imaging system, and the quantitative data shown represent wound closure at the 24 h endpoint. Data represent the mean ± SEM of three independent experiments. ** *p* < 0.01 vs. control group.

**Table 1:** ^1^H (800 MHz) and ^13^C (200 MHz) NMR data of compounds **1**–**3** in CD_3_OD *^a^*.

Position	1		Position	2		3	
	*δ*_H_ (*J* in Hz)	*δ*_C_		*δ*_H_ (*J* in Hz)	*δ*_C_	*δ*_H_ (*J* in Hz)	*δ*_C_
1			1		139.8		136.2
2	4.72 d (6.5)	81.1	2	6.72 s	103.3	6.77 s	103.5
3	4.02 m	67.3	3		154.6		154.6
4	2.83 dd (16.5, 5.0); 2.59 dd (16.5, 7.5)	26.2	4		135.1		140.1
5		155.7	5		154.6		154.6
6	6.22 d (2.0)	95.8	6	6.72 s	103.3	6.77 s	103.5
7		157.2	7	5.56 d (6.0)	88.5	5.57 d (5.5)	88.1
8	6.26 d (2.0)	95.1	8	3.46 m	55.4	3.44 (7.5, 5.5)	55.9
9		155.2	9	3.86 overlap; 3.76 dd (11.0, 7.5)	64.8	3.87 dd (11.0, 5.0); 3.75 m	65.1
10		102.0	1′		137.1		136.9
1′		130.6	2′	6.74 brs	113.9	6.58 brs	116.9
2′	6.86 d (2.0)	113.5	3′		145.1		141.9
3′		144.6	4′		147.3		146.1
4′		144.8	5′		129.3		129.2
5′	6.77 d (8.0)	114.6	6′	6.72 brs	117.7	6.59 brs	116.4
6′	6.75 dd (8.0, 2.0)	118.1	7′	2.63 m	32.6	2.56 m	32.5
1″	4.92 d (7.5)	100.5	8′	1.82 m	35.6	1.79 m	35.5
2″	3.47 m	73.1	9′	3.57 t (6.5)	61.9	3.55 t (6.5)	62.1
3″	3.51 m	76.3	1″	5.21 d (1.5)	103.3	3.74 dd (5.0, 1.5)	61.9
4″	3.47 m	70.1	2″	4.11 dd (3.0, 1.5)	71.8	3.99 quint (5.0)	84.6
5″	3.79 m	73.9	3″	3.87 m	72.0	3.74 dd (5.0, 1.5)	61.9
6″	4.72 overlap; 4.36 dd (12.0, 6.5)	63.9	4″	3.42 t (10.0)	73.5		
7″		71.5	5″	4.29 dq (10.0, 6.5)	70.8		
8″		155.2	6″	1.20 d (6.0)	17.6		
9″	7.54 d (8.5)	124.0	3-OCH_3_	3.80 s	56.2	3.83 s	56.3
10″	8.00 d (8.5)	129.1	5-OCH_3_	3.80 s	56.2	3.83 s	56.3
11″		127.7	3′-OCH_3_	3.87 s	56.4		
12″	8.00 d (8.5)	129.1					
13″	7.54 d (8.5)	124.1					
14″		166.7					
15″	1.53 s	30.1					
16″	1.52 s	30.1					

*^a^* Assignments were based on COSY, HSQC, and HMBC. The ^13^C chemical shifts in compounds **1**–**3** were assigned from the carbon dimensions of the HSQC and HMBC spectra. Coupling constants (in Hz) are in parentheses.

## Data Availability

The data presented in this study are available within the article and its Supplementary Materials.

## Supplementary Materials

The following supporting information can be downloaded at https://www.mdpi.com/article/10.3390/ijms27198630/s1.
