# Supplementary material for: Polyphenols from the Root Barks of Ulmus davidiana var. japonica and Their Wound Healing Potential
Source: Int J Mol Sci. 2026 Sep 26;27(19):8630. doi: 10.3390/ijms27198630 (PMC13664828; doi:10.3390/ijms27198630)
Supplement: Supplementary file 1 [file ijms-27-08630-s001.zip › ijms-4507217-supplementary.pdf]

Polyphenols from the Root Barks of *Ulmus davidiana* var. *japonica* and Their Wound Healing Potential

Jae Sik Yu [1,2], Gakyung Lee [1], Sooyeon Kim [1], Yoon-Joo Ko [3], Min Cheol Kang [4], Sun Yeou Kim [4], Min Hye Yang [5,*] and Ki Hyun Kim [2,*]

[1] Department of Integrative Biological Sciences and Industry, Sejong University, 209 Neungdong-ro, Gwangjin-gu, Seoul 05006, Republic of Korea

[2] School of Pharmacy, Sungkyunkwan University, Suwon 16419, Republic of Korea

[3] Laboratory of Nuclear Magnetic Resonance, National Center for Inter-University Research Facilities (NCIRF), Seoul National University, Gwanak-gu, Seoul 08826, Republic of Korea

[4] College of Pharmacy, Gachon University, Incheon 21936, Republic of Korea

[5] Department of Pharmacy, College of Pharmacy and Research Institute for Drug Development, Pusan National University, Busan 46241, Republic of Korea

* Correspondence: mhyang@pusan.ac.kr (M.H.Y.); khkim83@skku.edu (K.H.K.)

Table of Contents

Figure S1. ^{1}H NMR spectrum of compound **1** in CD_3OD (800 MHz)………………………...S3

Figure S2. HSQC spectrum of compound **1** in CD_3OD……………………......................S3

Figure S3. HMBC spectrum of compound **1** in CD_3OD……………………......................S4

Figure S4. COSY spectrum of compound **1** in CD_3OD……………………......................S4

Figure S5. NOESY spectrum of compound **1** in CD_3OD……………………....................S5

Figure S6. HRESIMS spectrum of compound **1**……………………..................................S5

Figure S7. UV spectrum of compound **1**……………………...S6

Figure S8. Experimental ECD spectrum of compound **1**……………………......................S6

Figure S9. Retention time of reaction products of thiocarbamoyl-thiazolidine derivatives of D-glucopyranose standard and glucopyranose from compound **1** in LC/MS analysis. ………….S7

Figure S10. Experimental ECD spectrum of aglycone of compound **1**………………………S7

Figure S11. ^{1}H NMR spectrum of compound **2** in CD_3OD (800 MHz) ………………………S8

Figure S12. HSQC spectrum of compound **2** in CD_3OD……………………………………S8

Figure S13. HMBC spectrum of compound **2** in CD_3OD……………………………………..S9

Figure S14. COSY spectrum of compound **2** in CD3OD………………………………..……S9

Figure S15. NOESY spectrum of compound **2** in CD3OD…………………………………S10

Figure S16. HRESIMS spectrum of compound **2**…………………………………………...S10

Figure S17. UV spectrum of compound **2**…………………………………………………...S11

Figure S18. Experimental ECD spectrum of compound **2**…………………………………...S11

Figure S19. Retention time of reaction products of thiocarbamoyl-thiazolidine derivatives of L-rhamnopyranose standard and rhamnopyranose from compound **2** in LC/MS analysis…..S12

Figure S20. Experimental ECD spectrum of aglycone of compound **2**……………………...S12

Figure S21. ^{1}H NMR spectrum of compound **3** in CD_3OD (800 MHz) ……………………..S13

Figure S22. HSQC spectrum of compound **3** in CD_3OD……………………………………S13

Figure S23. HMBC spectrum of compound **3** in CD_3OD…………………………………....S14

Figure S24. COSY spectrum of compound **3** in CD3OD……………………...……….S14

Figure S25. NOESY spectrum of compound **3** in CD3OD…………………………………..S15

Figure S26. HRESIMS spectrum of compound **3**…………………………………………...S15

Figure S27. UV spectrum of compound **3**…………………………….........................S16

Figure S28. Experimental ECD spectrum of compound **3**…………………………………..S16

Figure S29. Effects of compounds **1–13** on the viability of HaCaT cells …………………S17

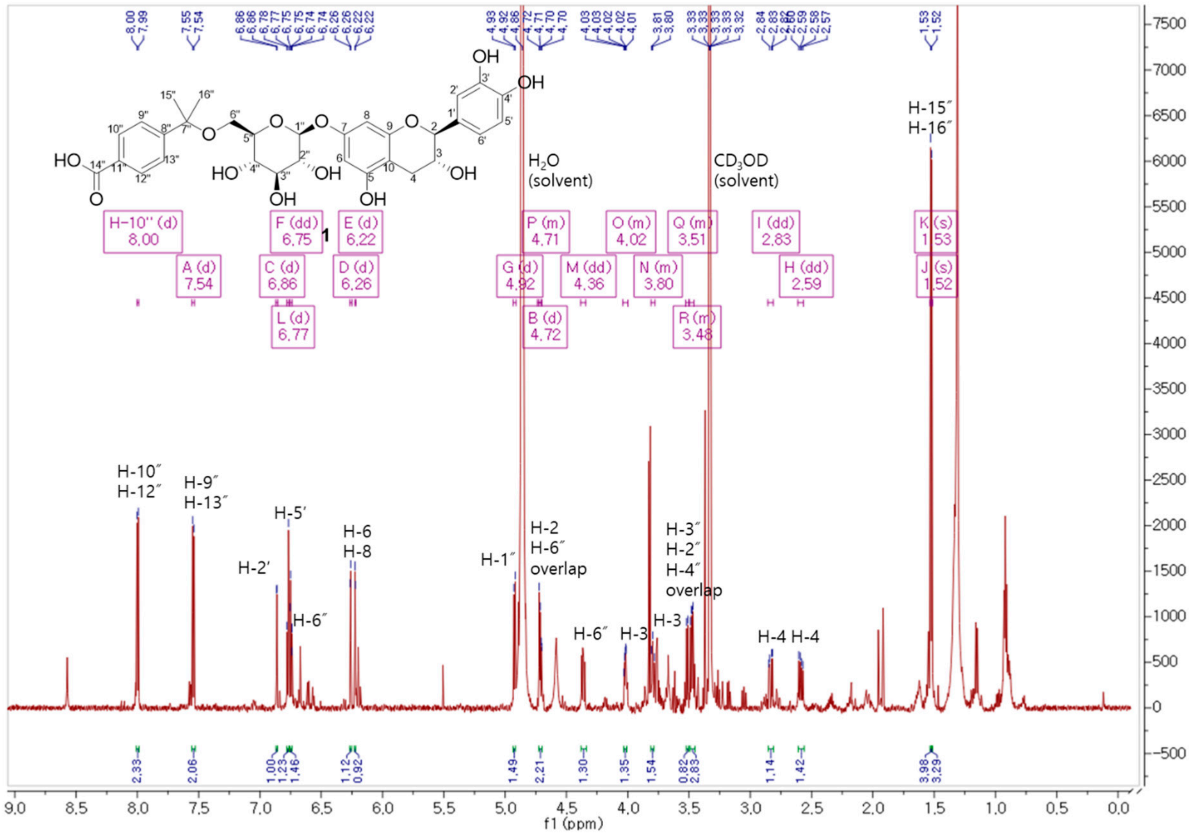

Figure S1. ^{1}H NMR spectrum of compound **1** in CD_3OD (800 MHz)

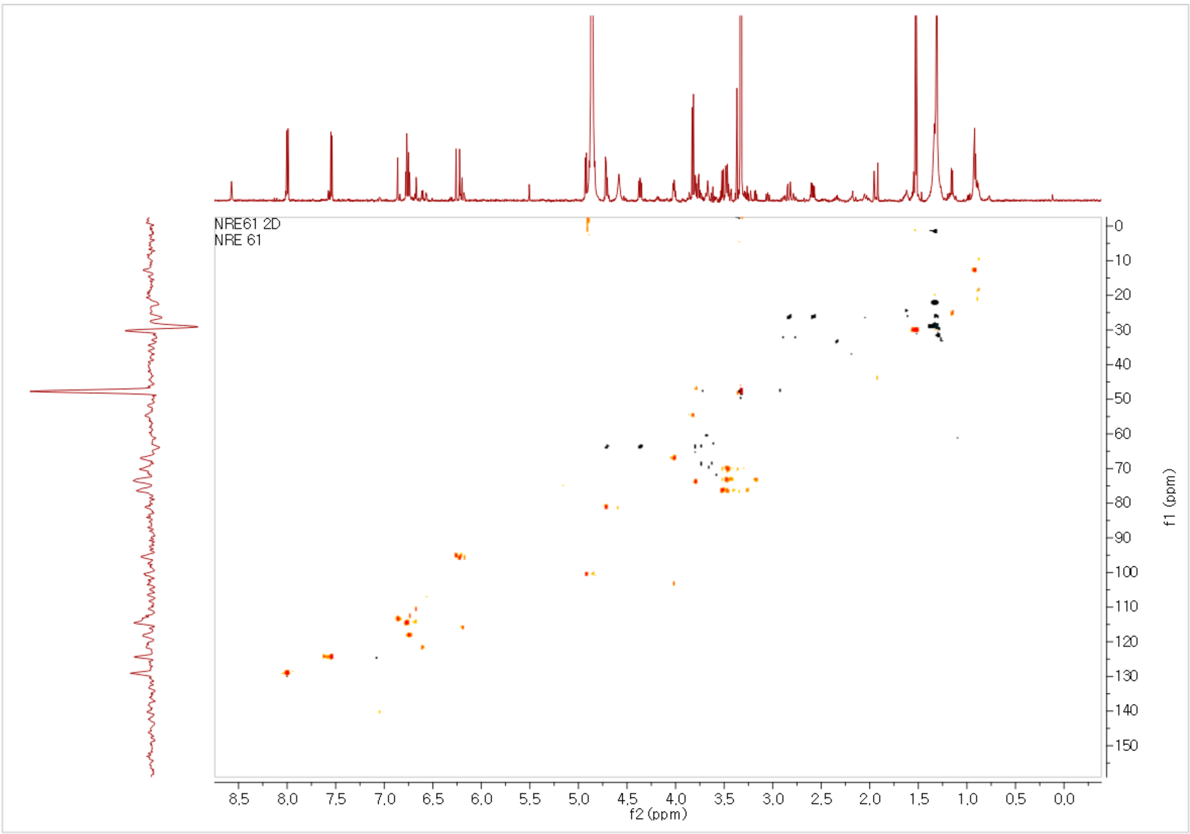

Figure S2. HSQC spectrum of compound **1** in CD_3OD. CH and CH_3 correlations are shown in red, whereas CH_2 correlations are shown in black.

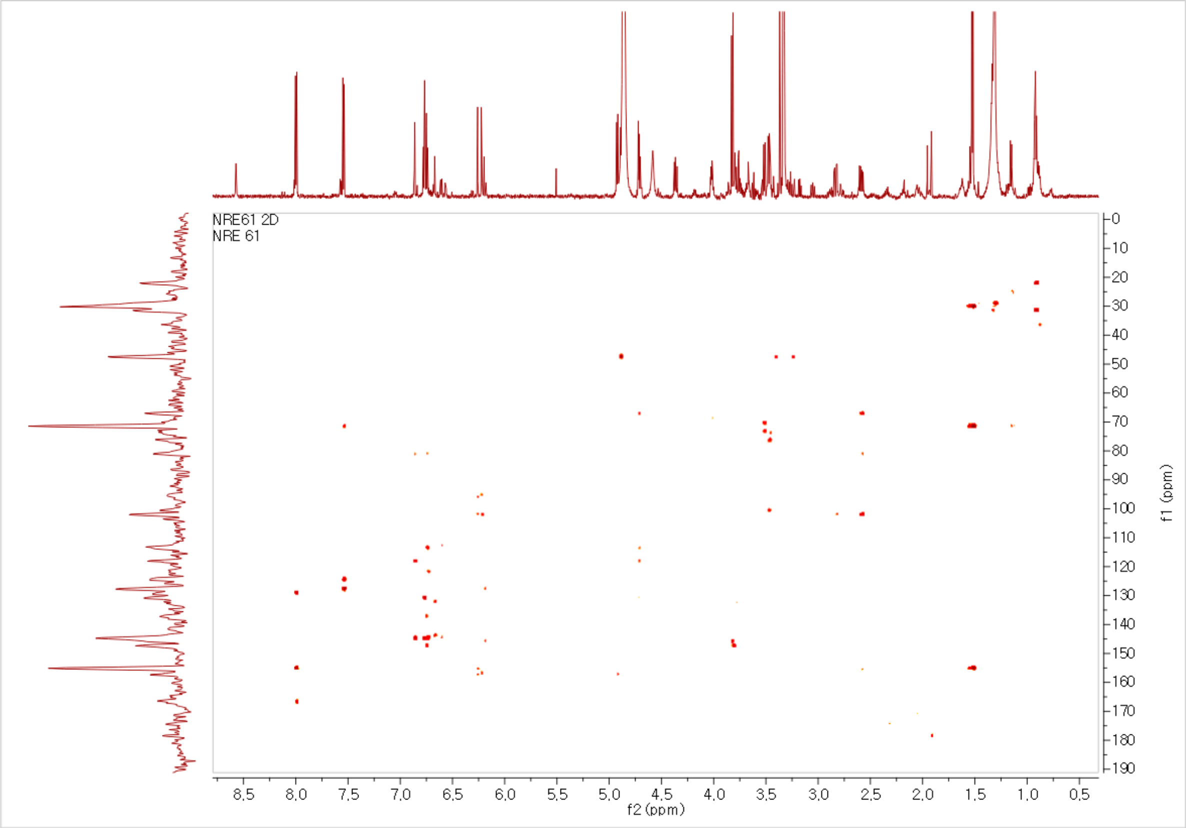

Figure S3. HMBC spectrum of compound **1** in CD_3OD

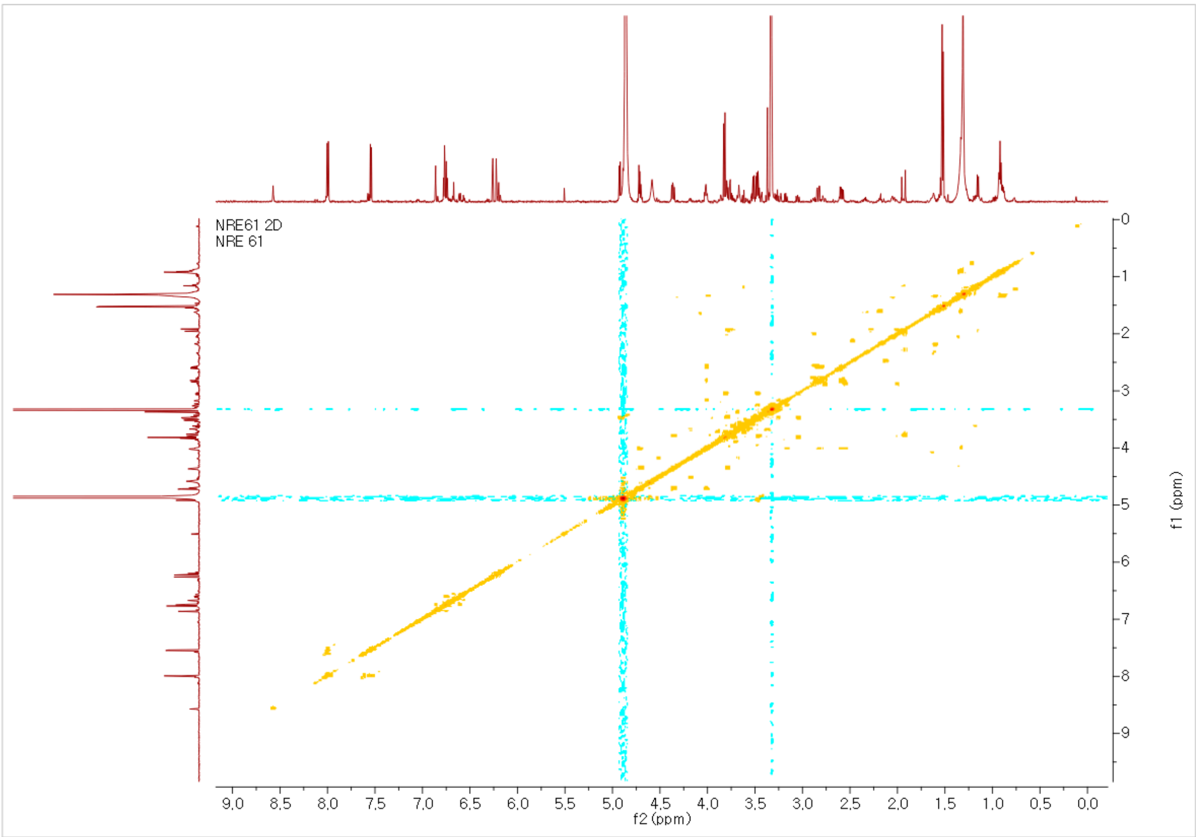

Figure S4. COSY spectrum of compound **1** in CD_3OD

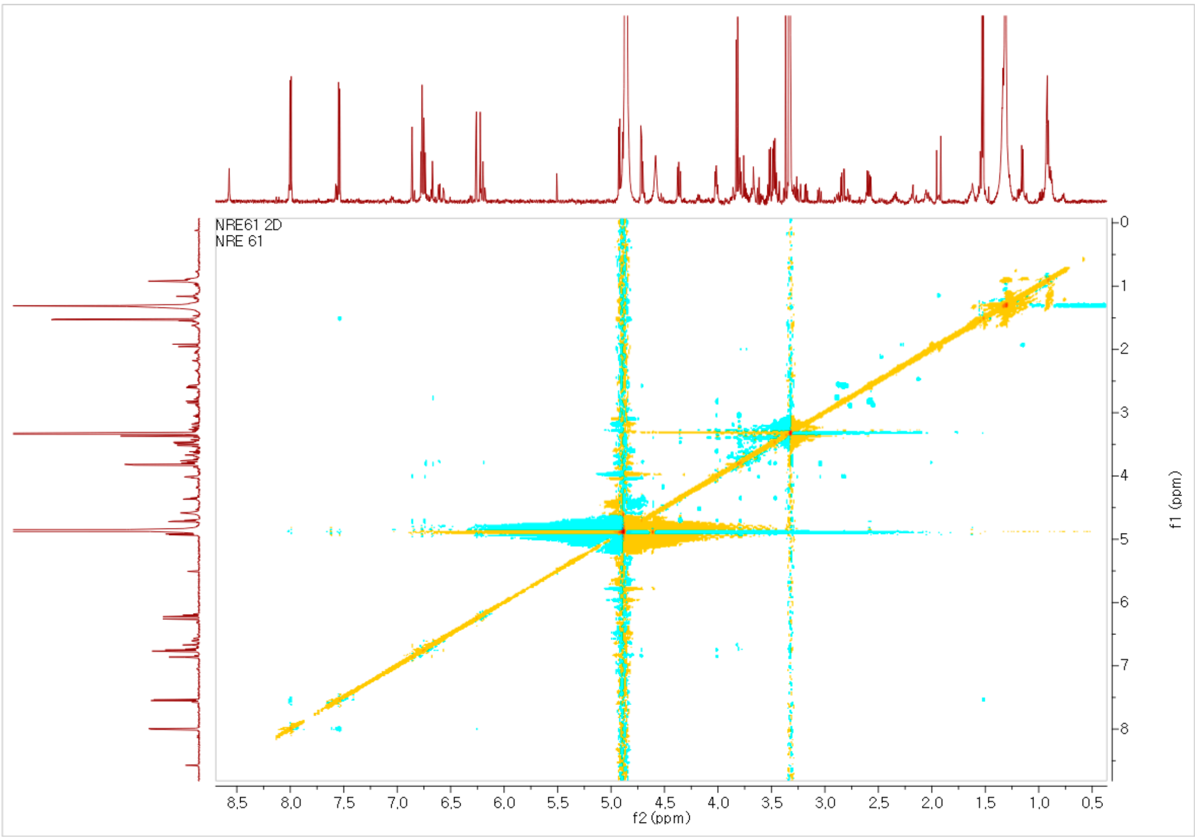

Figure S5. NOESY spectrum of compound **1** in CD_3OD

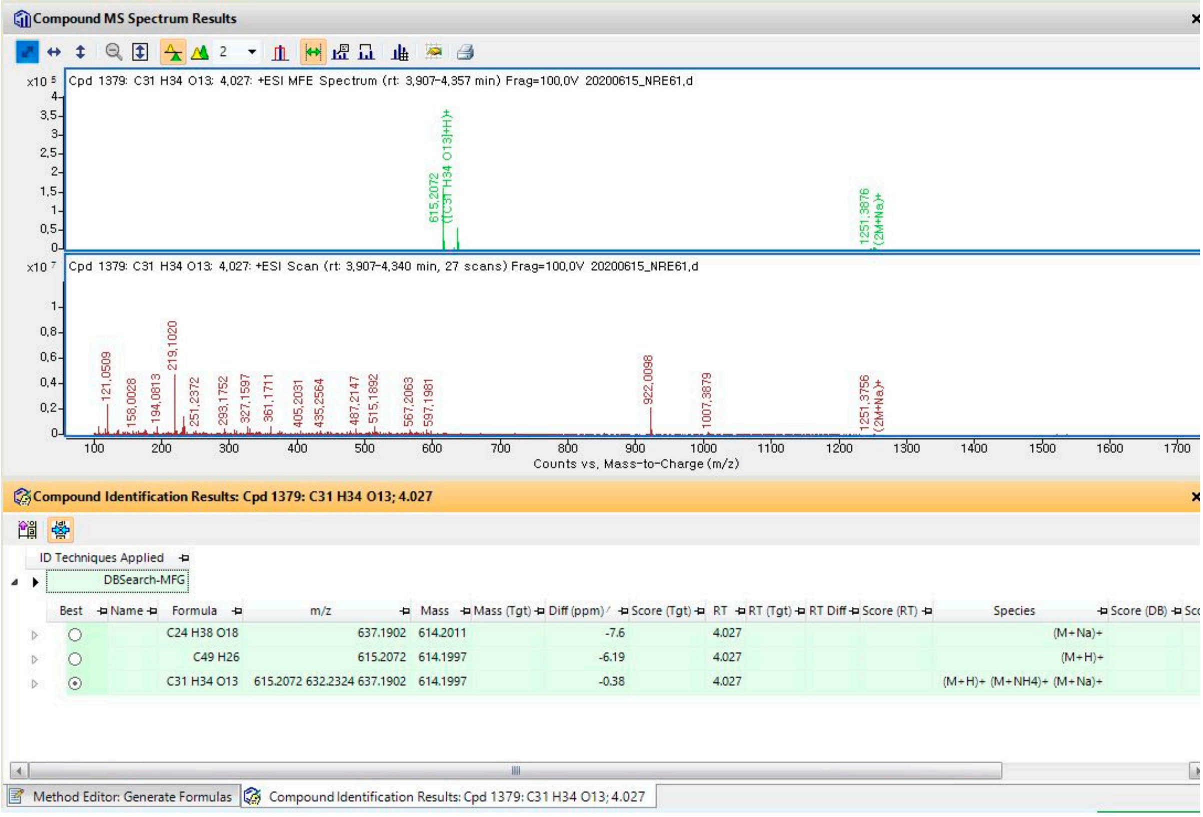

Figure S6. HRESIMS spectrum of compound **1**

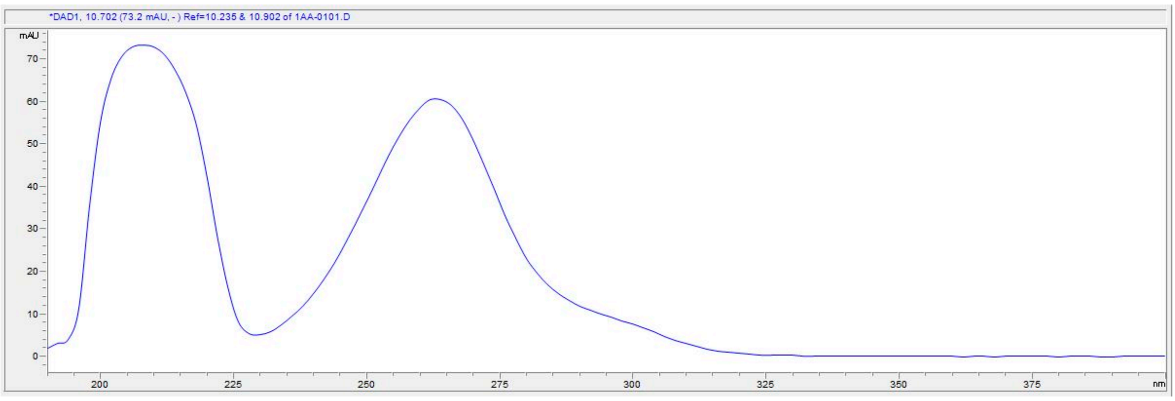

Figure S7. UV spectrum of compound **1**

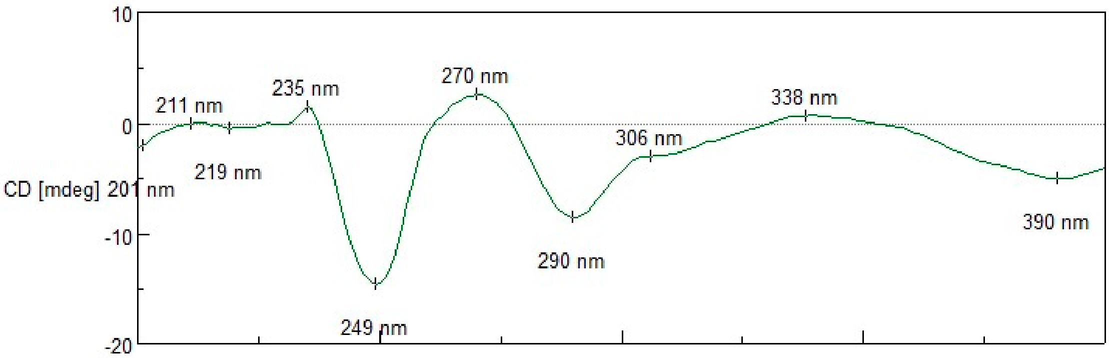

Figure S8. Experimental ECD spectrum of compound **1**

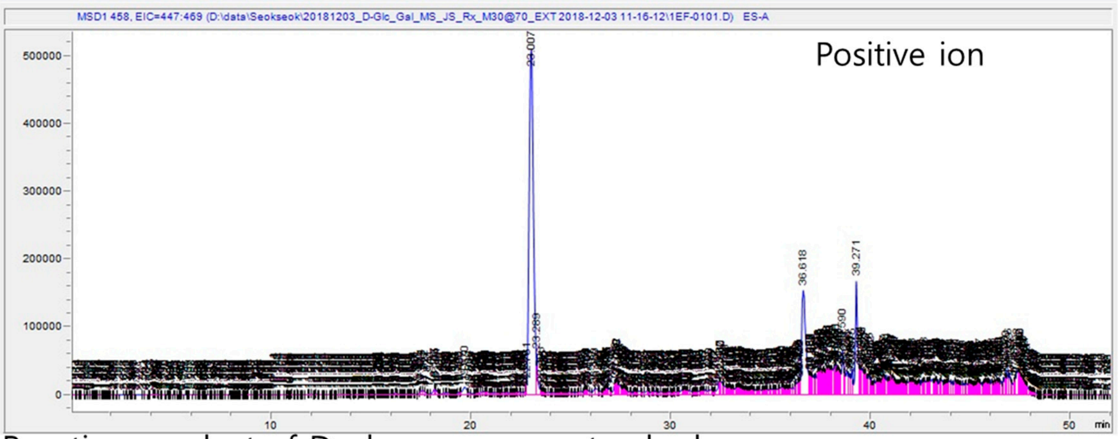

Reaction product of D-glucopyranose standard

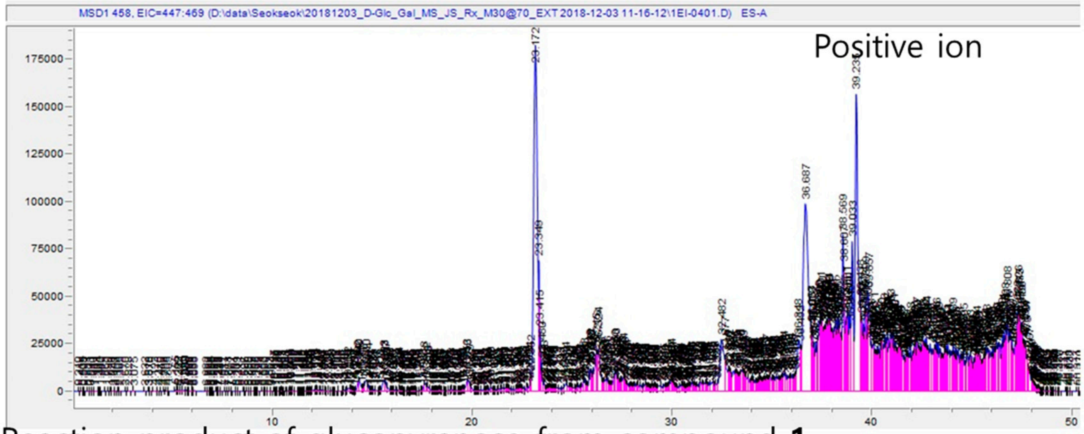

Reaction product of glucopyranose from compound **1**

Figure S9. Retention time of reaction products of thiocarbamoyl-thiazolidine derivatives of D-glucopyranose standard and glucopyranose from compound **1** in LC/MS analysis.

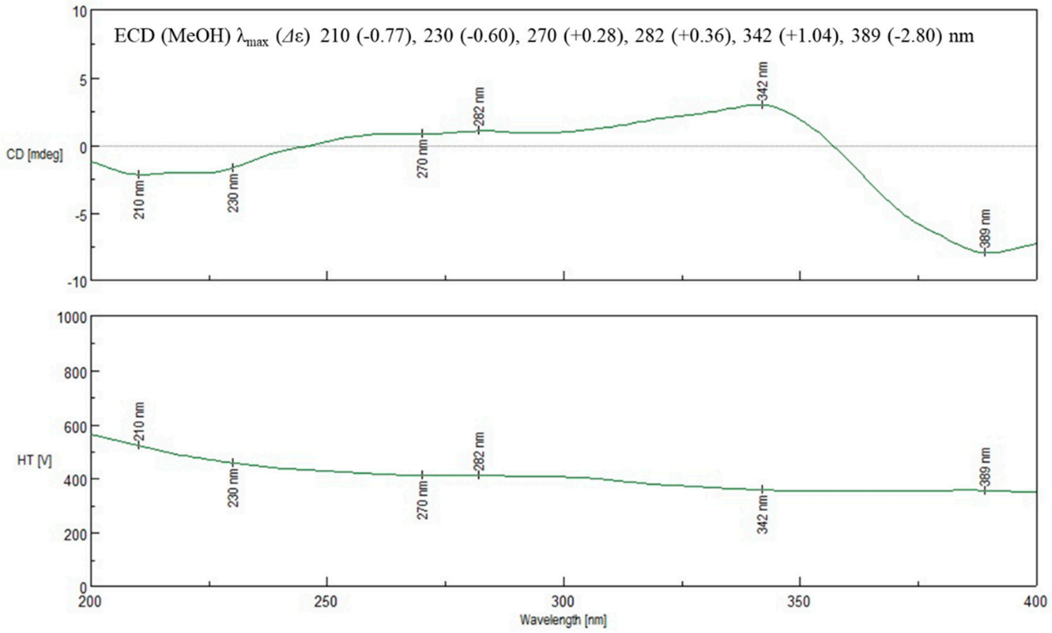

Figure S10. Experimental ECD spectrum of aglycone of compound **1**

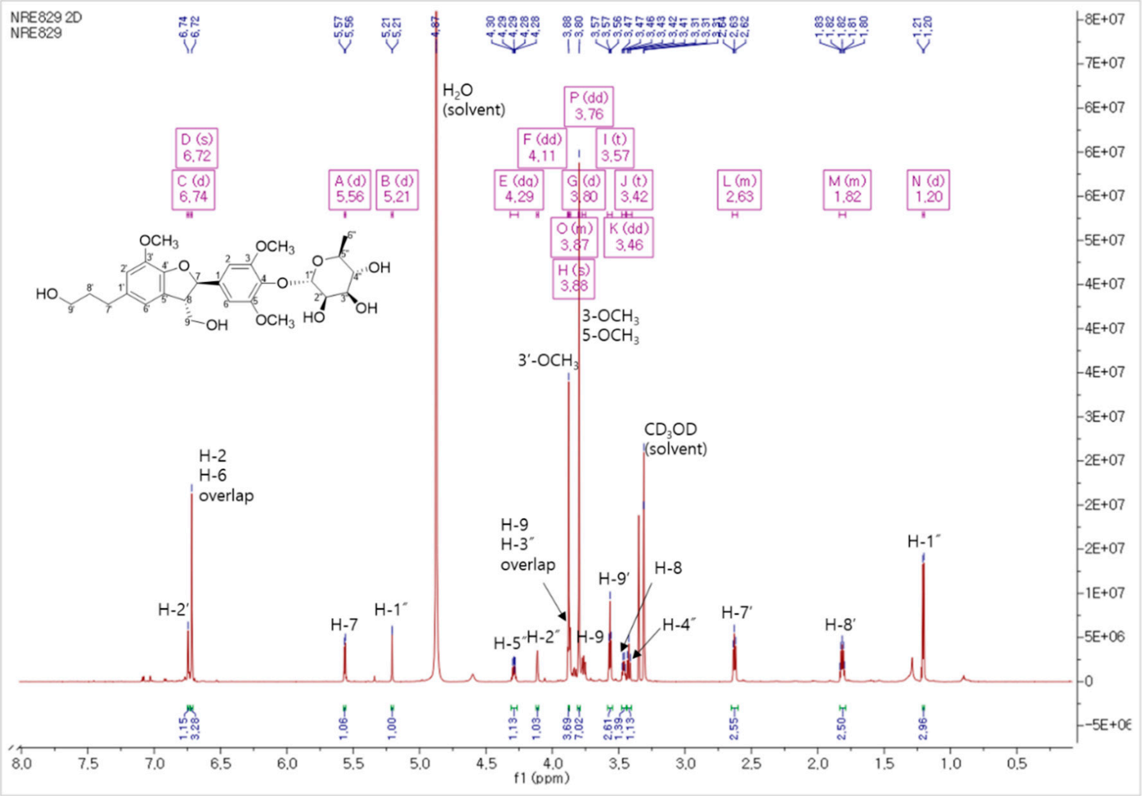

Figure S11. ^{1}H NMR spectrum of compound **2** in CD_3OD (800 MHz)

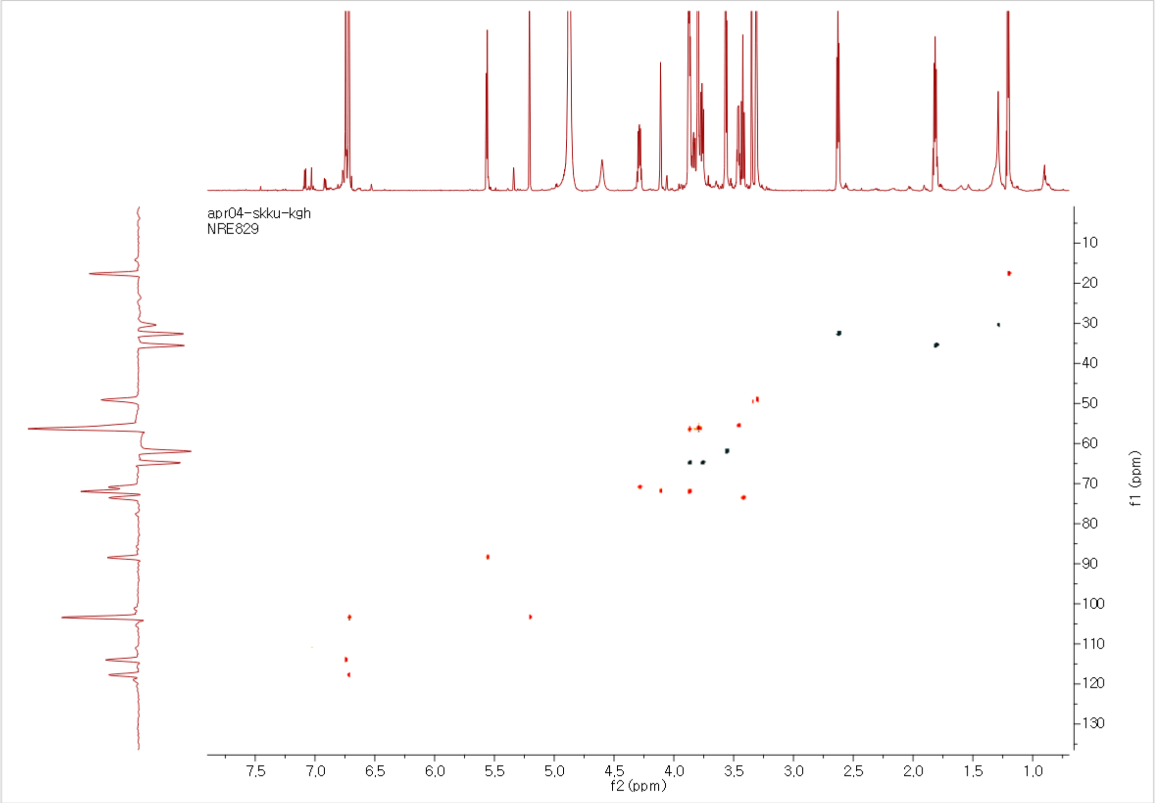

Figure S12. HSQC spectrum of compound **2** in CD_3OD. CH and CH_3 correlations are shown in red, whereas CH_2 correlations are shown in black.

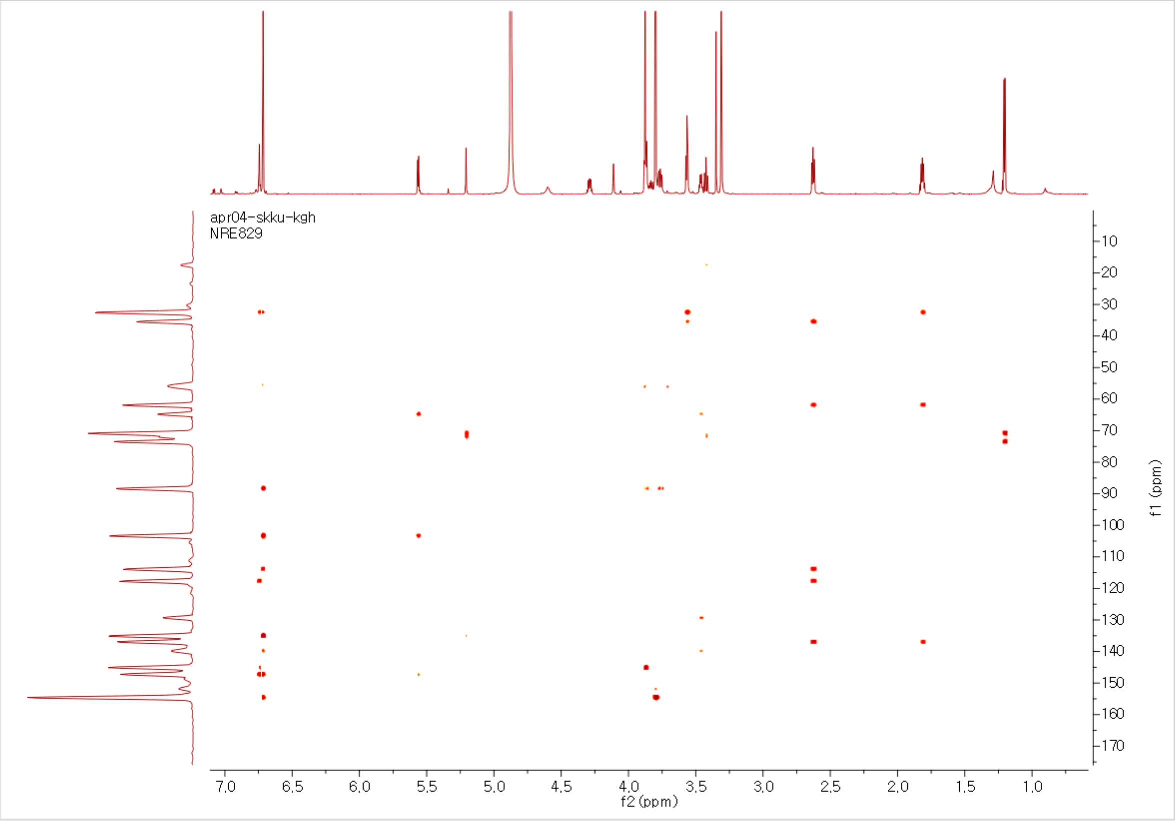

Figure S13. HMBC spectrum of compound **2** in CD_3OD

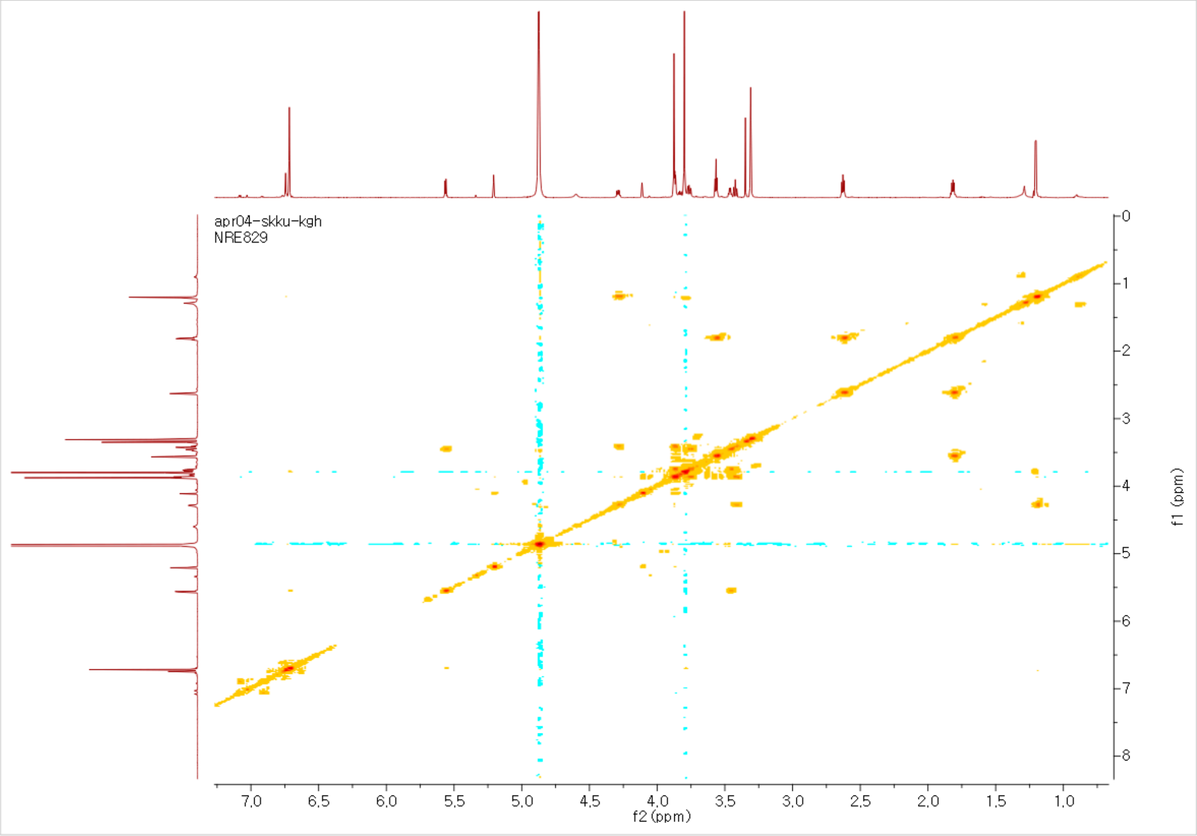

Figure S14. COSY spectrum of compound **2** in CD_3OD

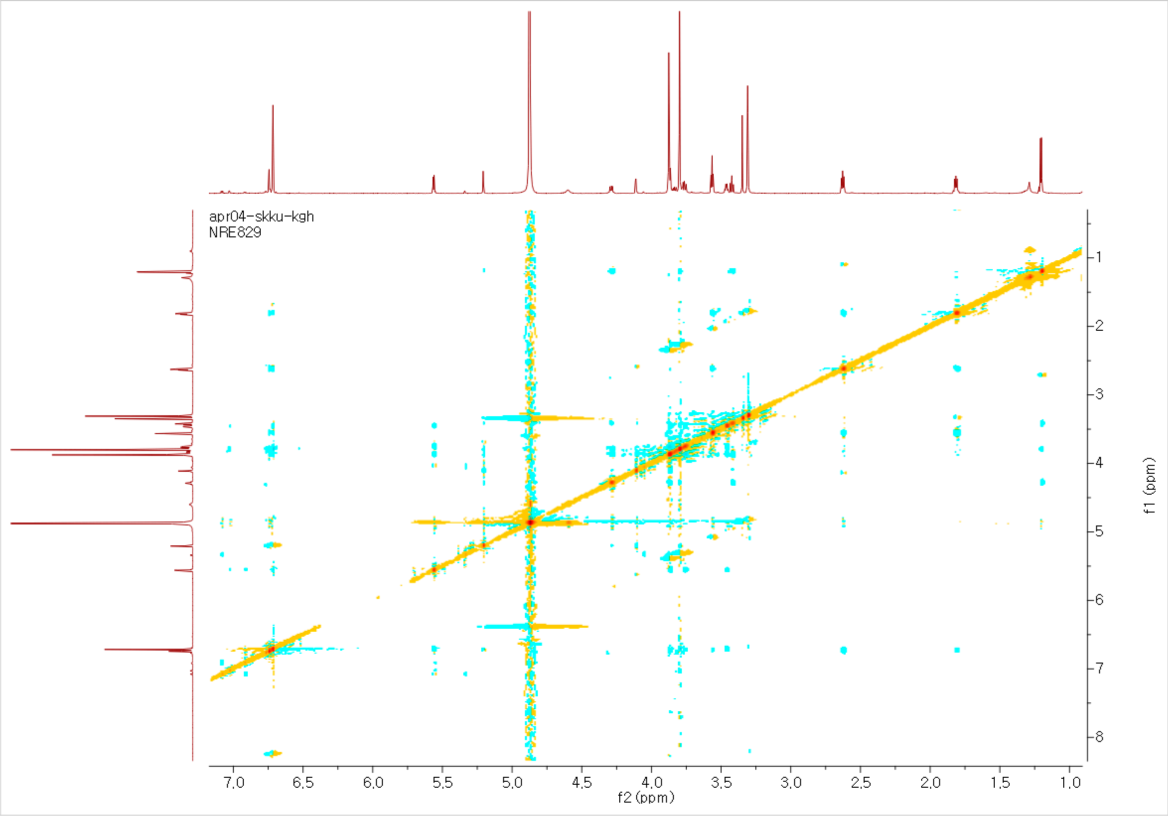

Figure S15. NOESY spectrum of compound **2** in CD_3OD

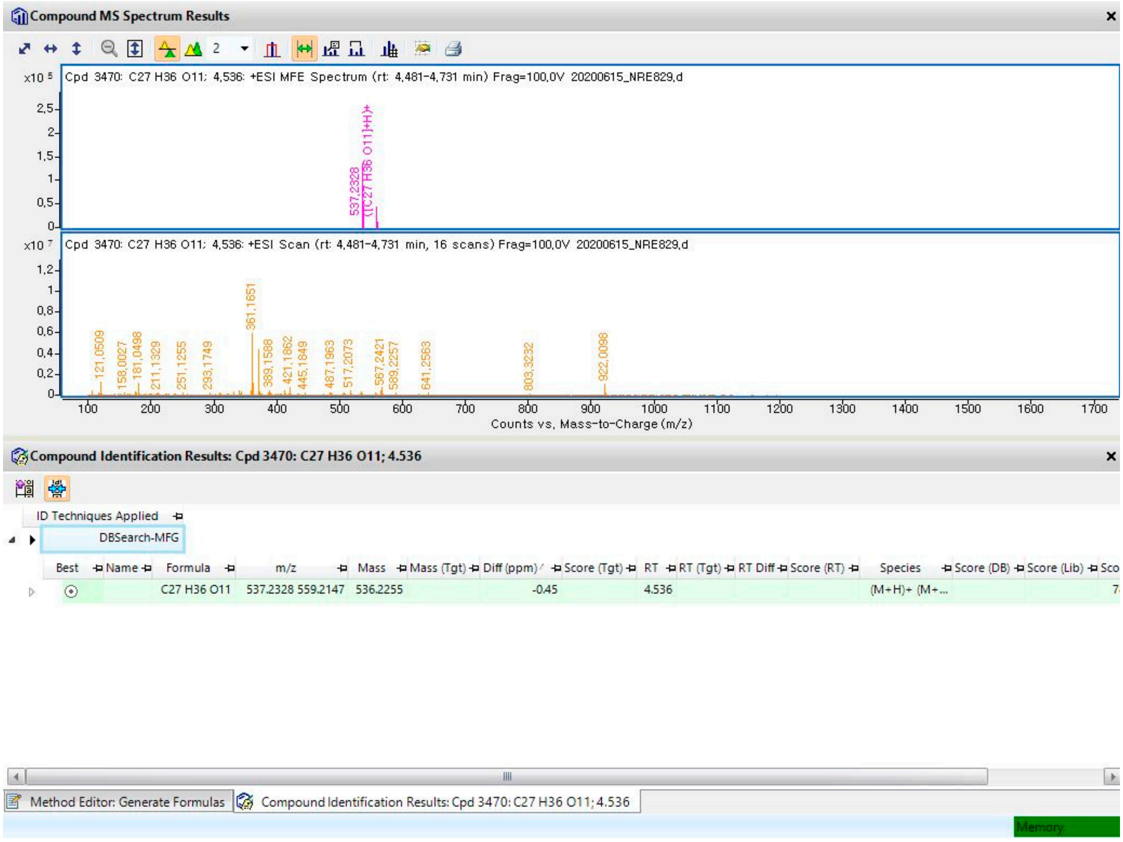

Figure S16. HRESIMS spectrum of compound **2**

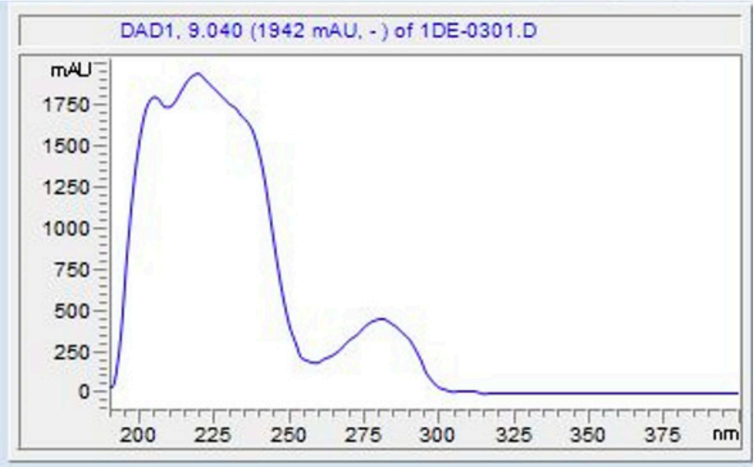

Figure S17. UV spectrum of compound **2**

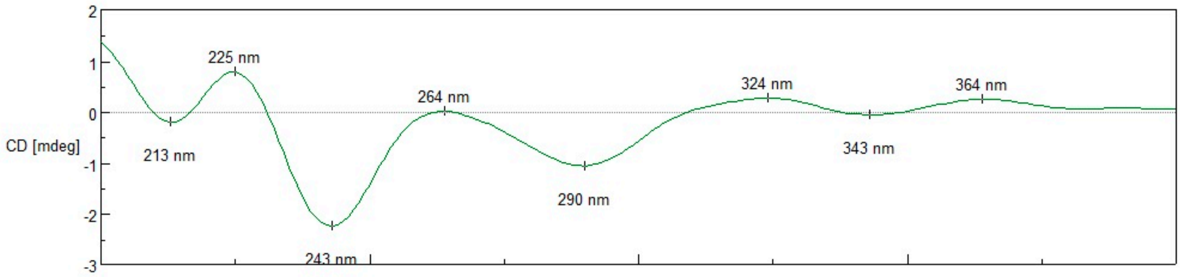

Figure S18. Experimental ECD spectrum of compound **2**

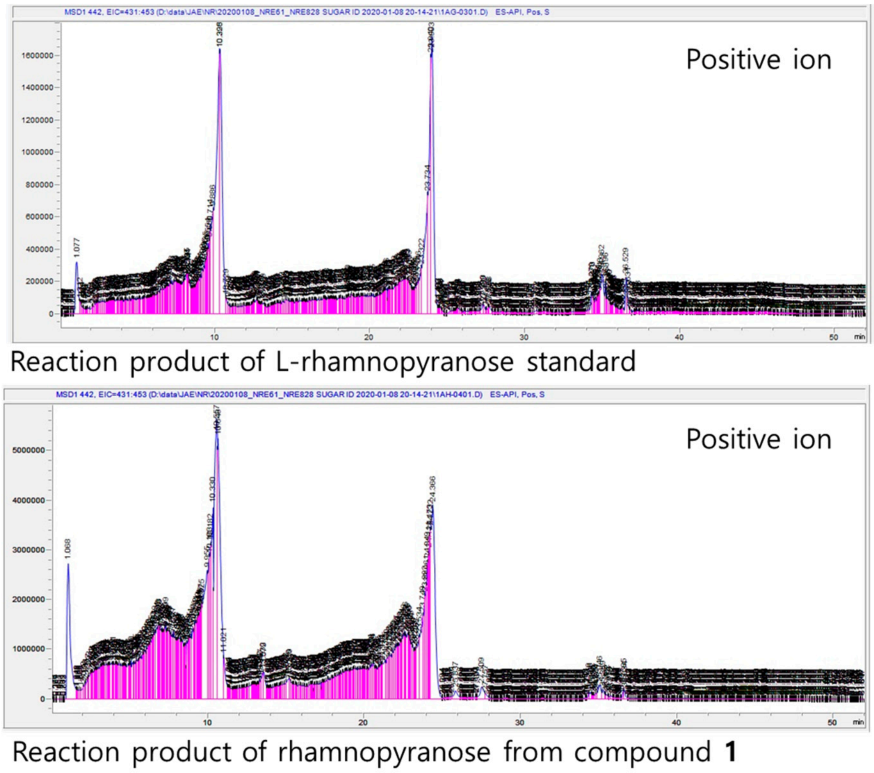

Figure S19. Retention time of reaction products of thiocarbamoyl-thiazolidine derivatives of L-rhamnopyranose standard and rhamnopyranose from compound **2** in LC/MS analysis.

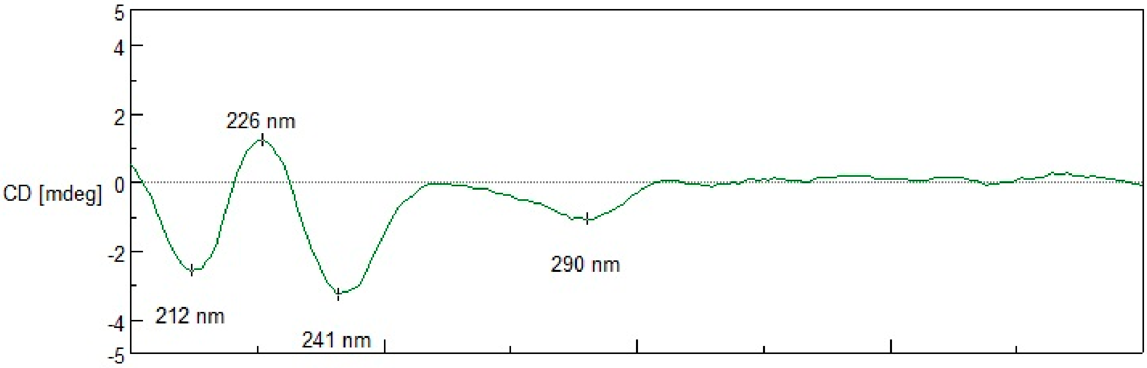

Figure S20. Experimental ECD spectrum of aglycone of compound **2**

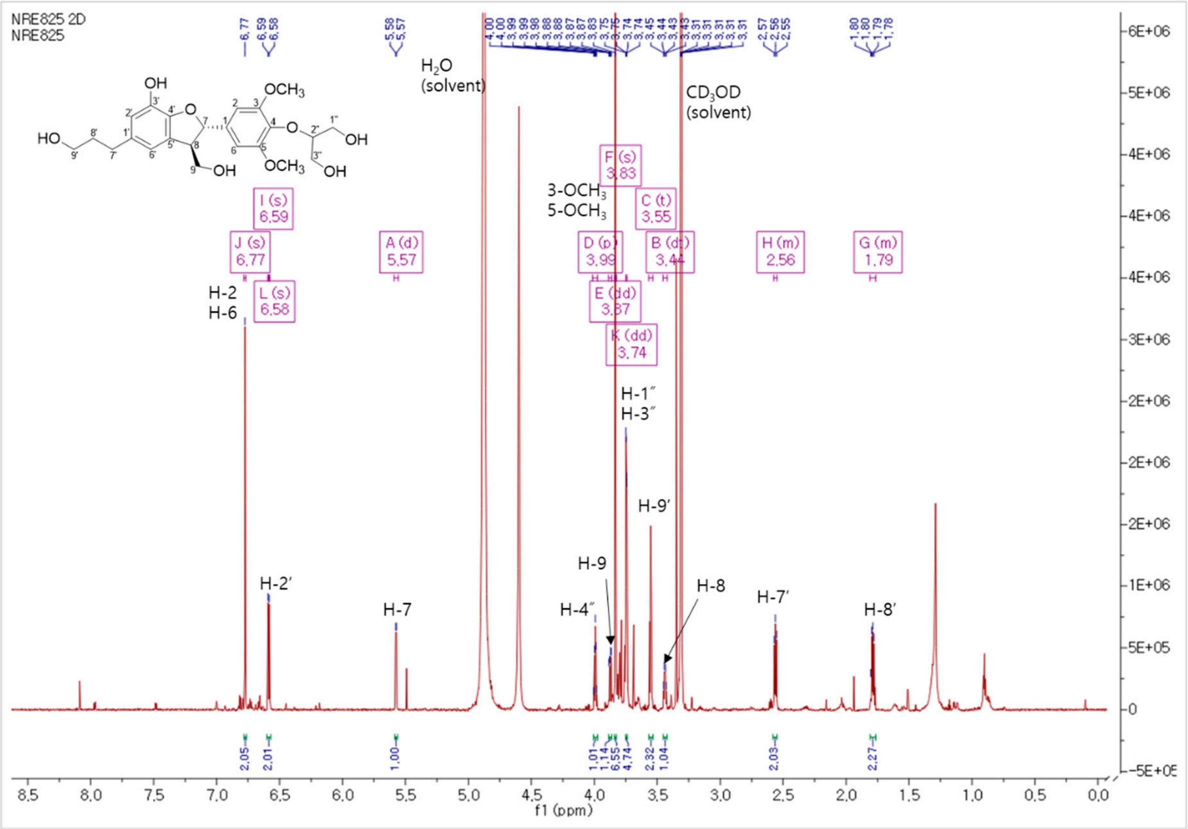

Figure S21. ^{1}H NMR spectrum of compound **3** in CD_3OD (800 MHz)

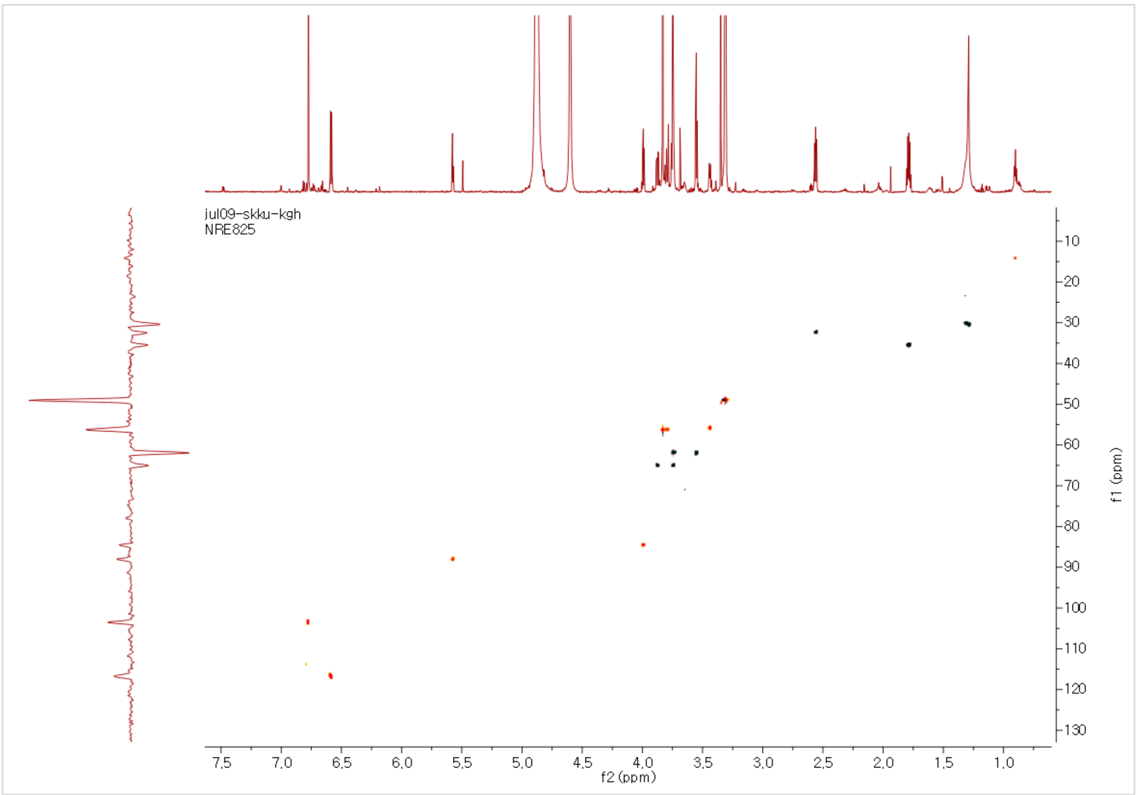

Figure S22. HSQC spectrum of compound **3** in CD_3OD. CH and CH_3 correlations are shown in red, whereas CH_2 correlations are shown in black.

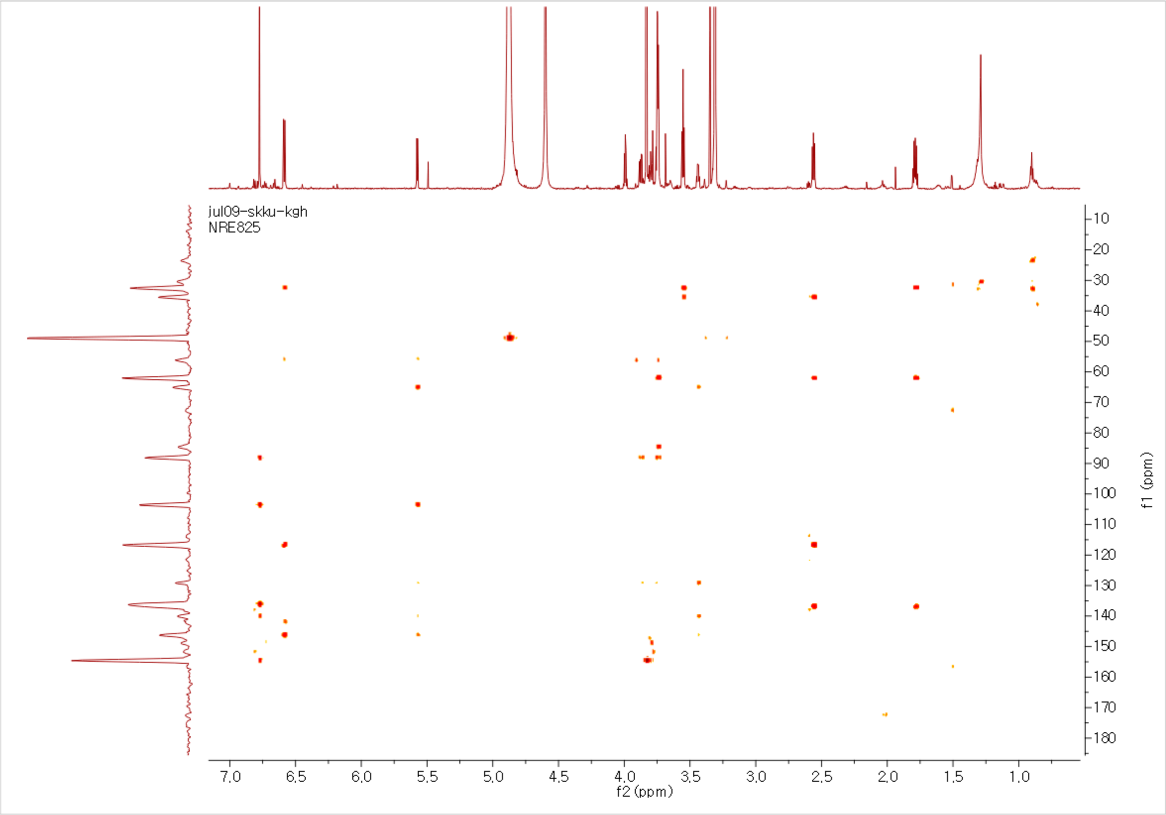

Figure S23. HMBC spectrum of compound **3** in CD_3OD

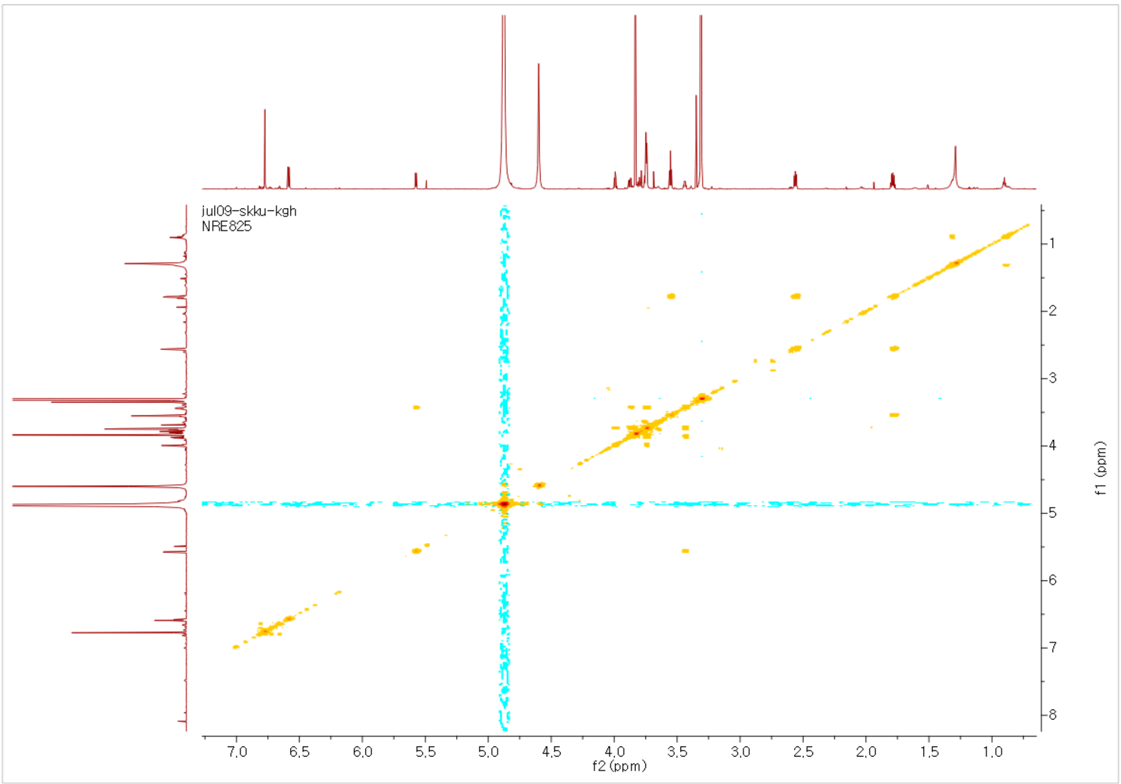

Figure S24. COSY spectrum of compound **3** in CD_3OD

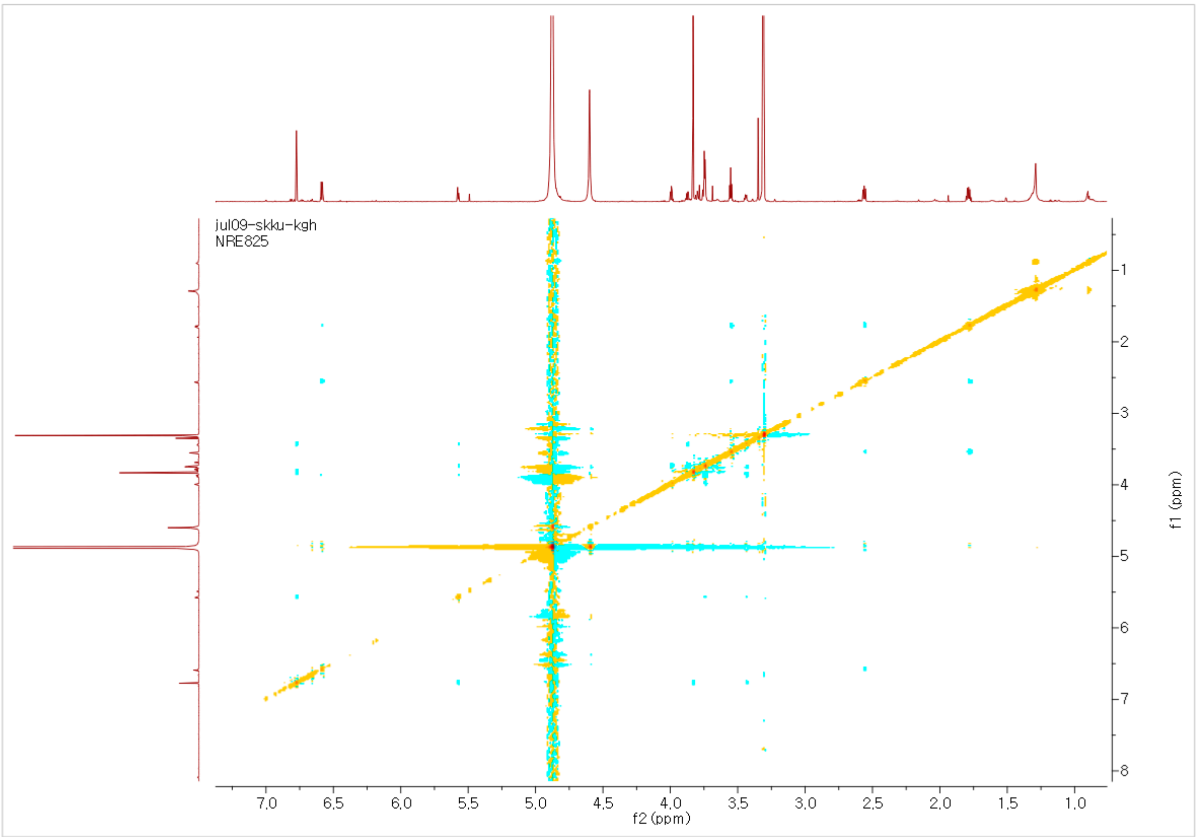

Figure S25. NOESY spectrum of compound **3** in CD_3OD

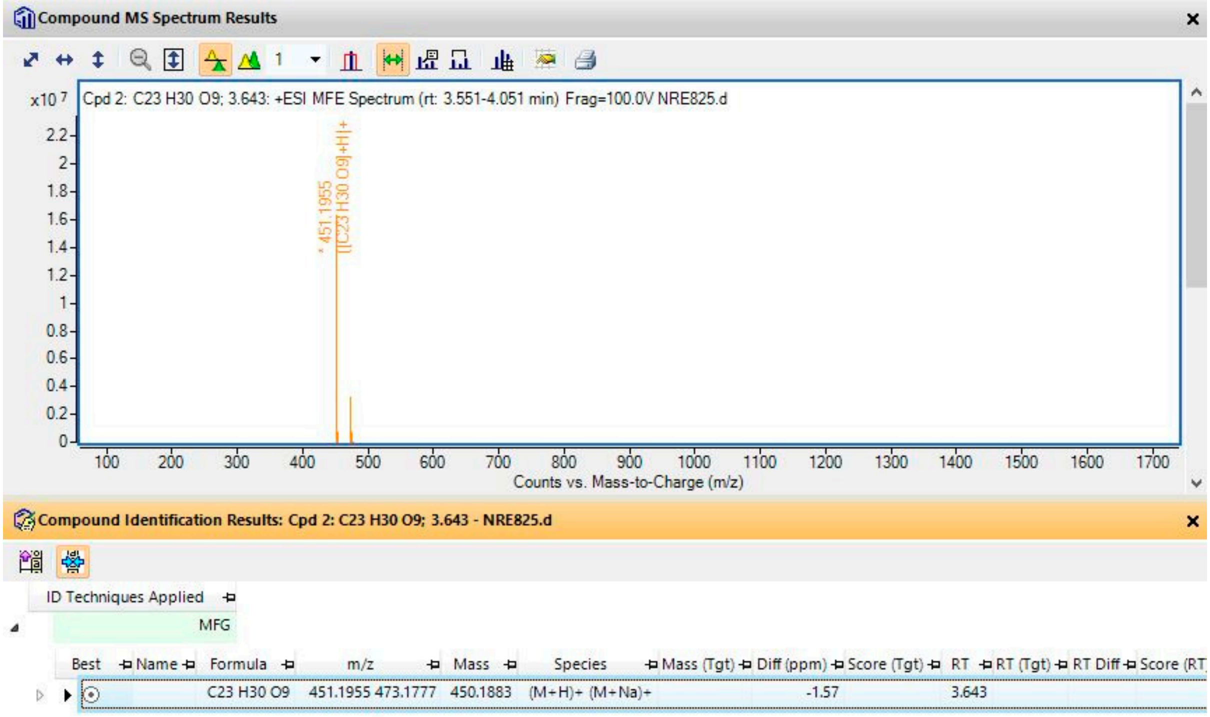

Figure S26. HRESIMS spectrum of compound **3**

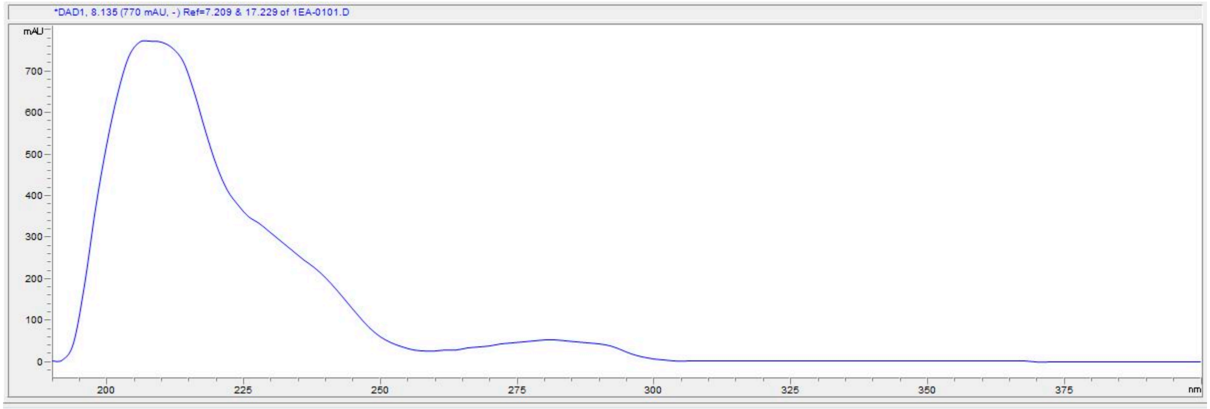

Figure S27. UV spectrum of compound **3**

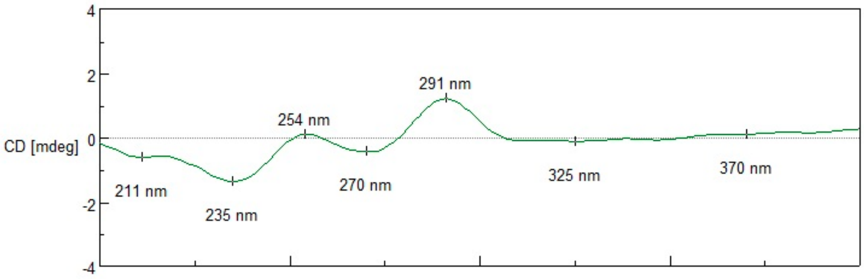

Figure S28. Experimental ECD spectrum of compound **3**

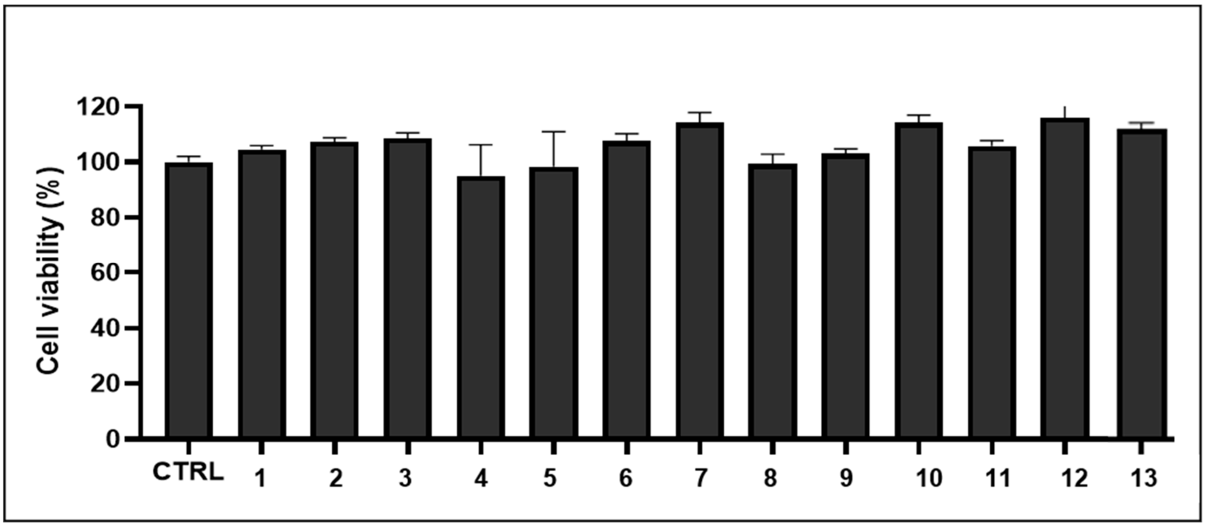

Figure S29. Effects of compounds **1–13** on the viability of HaCaT cells. HaCaT cells were treated with each compound at 10 μM for 24 h, and cell viability was determined using the MTT assay. Data are presented as the mean ± SEM of three independent experiments.
